# A mutant-based analysis of the establishment of Nod-independent symbiosis in the legume *Aeschynomene evenia*

**DOI:** 10.1093/plphys/kiac325

**Published:** 2022-07-25

**Authors:** Johan Quilbé, Nico Nouwen, Marjorie Pervent, Rémi Guyonnet, Julie Cullimore, Frédéric Gressent, Natasha Horta Araújo, Djamel Gully, Christophe Klopp, Eric Giraud, Jean-François Arrighi

**Affiliations:** IRD, Laboratoire des Symbioses Tropicales et Méditerranéennes (LSTM), UMR IRD/SupAgro/INRAE/UM/CIRAD, TA-A82/J-Campus de Baillarguet, Montpellier 34398, France; IRD, Plant Health Institute of Montpellier (PHIM), UMR IRD/SupAgro/INRAE/UM/CIRAD, TA-A82/J – Campus de Baillarguet, Montpellier 34398, France; IRD, Laboratoire des Symbioses Tropicales et Méditerranéennes (LSTM), UMR IRD/SupAgro/INRAE/UM/CIRAD, TA-A82/J-Campus de Baillarguet, Montpellier 34398, France; IRD, Plant Health Institute of Montpellier (PHIM), UMR IRD/SupAgro/INRAE/UM/CIRAD, TA-A82/J – Campus de Baillarguet, Montpellier 34398, France; IRD, Laboratoire des Symbioses Tropicales et Méditerranéennes (LSTM), UMR IRD/SupAgro/INRAE/UM/CIRAD, TA-A82/J-Campus de Baillarguet, Montpellier 34398, France; IRD, Plant Health Institute of Montpellier (PHIM), UMR IRD/SupAgro/INRAE/UM/CIRAD, TA-A82/J – Campus de Baillarguet, Montpellier 34398, France; IRD, Laboratoire des Symbioses Tropicales et Méditerranéennes (LSTM), UMR IRD/SupAgro/INRAE/UM/CIRAD, TA-A82/J-Campus de Baillarguet, Montpellier 34398, France; Laboratory of Plant-Microbe Interactions and Environment (LIPME), University Toulouse III, INRAE, CNRS, Castanet-Tolosan, France; IRD, Laboratoire des Symbioses Tropicales et Méditerranéennes (LSTM), UMR IRD/SupAgro/INRAE/UM/CIRAD, TA-A82/J-Campus de Baillarguet, Montpellier 34398, France; IRD, Plant Health Institute of Montpellier (PHIM), UMR IRD/SupAgro/INRAE/UM/CIRAD, TA-A82/J – Campus de Baillarguet, Montpellier 34398, France; IRD, Laboratoire des Symbioses Tropicales et Méditerranéennes (LSTM), UMR IRD/SupAgro/INRAE/UM/CIRAD, TA-A82/J-Campus de Baillarguet, Montpellier 34398, France; IRD, Plant Health Institute of Montpellier (PHIM), UMR IRD/SupAgro/INRAE/UM/CIRAD, TA-A82/J – Campus de Baillarguet, Montpellier 34398, France; IRD, Laboratoire des Symbioses Tropicales et Méditerranéennes (LSTM), UMR IRD/SupAgro/INRAE/UM/CIRAD, TA-A82/J-Campus de Baillarguet, Montpellier 34398, France; IRD, Plant Health Institute of Montpellier (PHIM), UMR IRD/SupAgro/INRAE/UM/CIRAD, TA-A82/J – Campus de Baillarguet, Montpellier 34398, France; Plateforme Bioinformatique Genotoul, BioinfoMics, UR875 Biométrie et Intelligence Artificielle, INRAE, Castanet-Tolosan, France; IRD, Laboratoire des Symbioses Tropicales et Méditerranéennes (LSTM), UMR IRD/SupAgro/INRAE/UM/CIRAD, TA-A82/J-Campus de Baillarguet, Montpellier 34398, France; IRD, Plant Health Institute of Montpellier (PHIM), UMR IRD/SupAgro/INRAE/UM/CIRAD, TA-A82/J – Campus de Baillarguet, Montpellier 34398, France; IRD, Laboratoire des Symbioses Tropicales et Méditerranéennes (LSTM), UMR IRD/SupAgro/INRAE/UM/CIRAD, TA-A82/J-Campus de Baillarguet, Montpellier 34398, France; IRD, Plant Health Institute of Montpellier (PHIM), UMR IRD/SupAgro/INRAE/UM/CIRAD, TA-A82/J – Campus de Baillarguet, Montpellier 34398, France

## Abstract

Intensive research on nitrogen-fixing symbiosis in two model legumes has uncovered the molecular mechanisms, whereby rhizobial Nod factors activate a plant symbiotic signaling pathway that controls infection and nodule organogenesis. In contrast, the so-called Nod-independent symbiosis found between *Aeschynomene evenia* and photosynthetic bradyrhizobia, which does not involve Nod factor recognition nor infection thread formation, is less well known. To gain knowledge on how Nod-independent symbiosis is established, we conducted a phenotypic and molecular characterization of *A. evenia* lines carrying mutations in different nodulation genes. Besides investigating the effect of the mutations on rhizobial symbiosis, we examined their consequences on mycorrhizal symbiosis and in nonsymbiotic conditions. Analyzing allelic mutant series for *AePOLLUX*, *Ca^2+^/calmodulin dependent kinase*, *AeCYCLOPS*, *nodulation signaling pathway 2 (AeNSP2*), and *nodule inception* demonstrated that these genes intervene at several stages of intercellular infection and during bacterial accommodation. We provide evidence that *AeNSP2* has an additional nitrogen-dependent regulatory function in the formation of axillary root hairs at lateral root bases, which are rhizobia-colonized infection sites. Our investigation of the recently discovered symbiotic actor *cysteine-rich receptor-like kinase* specified that it is not involved in mycorrhization; however, it is essential for both symbiotic signaling and early infection during nodulation. These findings provide important insights on the *modus operandi* of Nod-independent symbiosis and contribute to the general understanding of how rhizobial–legume symbioses are established by complementing the information acquired in model legumes.

## Teaser

Characterization of *Aeschynomene evenia* mutants altered in nodulation provides information on alternative mechanisms of rhizobium–legume symbiosis

## Introduction

Plants have developed different strategies to cope with nutrient deprivation, notably by adapting their development and metabolism. Another ingenious solution is to establish symbiotic interactions with soil-borne microorganisms to enhance nutrient uptake. At least 80% of land plants are capable of forming a symbiosis with Glomeromycota fungi called arbuscular mycorrhiza. In this symbiosis, fungal hyphae penetrate plant roots and form arbuscules inside cortical cells. These symbiotic structures are the interface for the uptake of inorganic phosphorus (P_i_) and other micronutrients used for plant nutrition. A limited number of flowering plants are also able to establish symbiosis with diazotrophic bacteria. Among these nitrogen-fixing symbioses, most members of the legume family (*Fabaceae*) interact with bacteria collectively known as rhizobia. These latter are hosted in a root-derived symbiotic organ, the nodule, where rhizobia reduce atmospheric nitrogen into ammonium to the plants’ benefit.

The rhizobium–legume symbiosis plays a prominent role in global biological nitrogen fixation, and legume crops are a major protein source for human and animal diets. Given their importance, intense research has been conducted on nodulation in legumes. Notably, forward genetic screens in two model legumes, barrel medic (*Medicago truncatula*) and *Lotus japonicus*, have identified numerous genes involved in the establishment of nodulation (Roy et al., 2020). It is now common knowledge that rhizobia produce lipochitooligosaccharidic Nod factors, which are perceived by plasma membrane-localized LysM-RLK receptors that act upstream of a Nod signaling pathway leading to the activation of a network of transcription factors in the cell nucleus. These latter coordinate the expression of genes involved in intracellular rhizobial infection via root hair infection threads, nodule organogenesis, and subsequent bacterial accommodation into nodules to fix nitrogen (Roy et al., 2020). These studies also provided visions on how nodulation could have evolved. First, several nodulation genes have also a nonsymbiotic function, suggesting that existing gene functions have been subsequently recruited for symbiosis (Liu et al., 2011). Second, many nodulation genes involved in signaling and infection appear to have been co-opted from roles in the more ancient mycorrhizal symbiosis (Gobbato, 2015; Radhakrishnan et al., 2020).

Research on *M. truncatula* and *L. japonicus* has boosted our understanding of nodulation mechanisms, but it is now also crystal clear that this knowledge is very model biased. The legume family is huge (approximately 20,000 species) and manifests a diversity of nodulation features (Masson-Boivin et al., 2009). In this respect, several *Aeschynomene* species are noticeable because they establish a symbiotic interaction with photosynthetic *Bradyrhizobium* strains without Nod factor recognition and infection thread formation (Giraud et al., 2007; Bonaldi et al., 2011). Whereas this so-called Nod-independent activation is singular in the legume family, rhizobial infection via intercellular penetration is observed in 25% of the genera (Ibáñez et al., 2017). In Nod-independent *Aeschynomene* species, bradyrhizobia first massively colonize axillary root hairs present at the base of lateral roots and then they progress intercellularly in the root cortex. A few cortical cells are subsequently infected and divide repeatedly to give rise to the nodule (Bonaldi et al., 2011). This infection mode is proposed to be mechanistically simpler than the one involving infection thread formation.

Deciphering the molecular basis of the Nod-independent symbiosis is expected to provide critical insights into the evolution and diversity of nodulation (Quilbé and Arrighi, 2021). To identify genes important for this distinctive symbiosis, the genome of *Aeschynomene evenia* was recently sequenced and a forward genetic approach was conducted on this species (Quilbé et al., 2021). Approximately 70,000 ethyl methanesulfonate (EMS)-mutagenized seedlings were screened for defects in nodulation. Sequencing of nodulation mutant plants led to the identification of several symbiotic genes, *AePOLLUX*, *Ca^2+^/calmodulin dependent kinase (AeCCaMK*), *AeCYCLOPS*, *nodulation signaling pathway 2 (AeNSP2*), and *nodule inception (AeNIN*), coding for known components of the Nod signaling pathway in model legumes, that acts downstream of the Nod factor receptors. The isolation of these *A. evenia* mutants provide the opportunity to explore the roles of these conserved signaling genes in a symbiotic context that differs from the well-studied model legumes. This mutant-based approach also led to the discovery of a symbiotic gene, *AeCRK*, encoding a cysteine-rich receptor-like kinase (CRK) (Quilbé et al., 2021). Interestingly, *AeCRK* putative orthologs are ancestral in legumes but absent in model legumes. Although *AeCRK* is required to trigger nodulation in *A. evenia*, its precise role is not yet known.

In this study, we aimed to better understand the roles of *AePOLLUX*, *AeCCaMK*, *AeCYCLOPS*, *AeNSP2*, *AeNIN*, and *AeCRK* in the establishment of the Nod-independent symbiosis. For this purpose, we undertook a detailed phenotypic and molecular characterization of allelic nodulation mutants altered in these genes in *A. evenia*. We found that the symbiotic signaling pathway intervenes at several steps of the establishment of the Nod-independent symbiosis and observed that, *AeNSP2* has an additional nonsymbiotic function to control the formation of axillary root hairs. We also obtained indications on how *AeCRK* can mediate activation of nodulation and evidence that, unlike most genes of the Nod signaling pathway, this gene probably does not intervene in arbuscular mycorrhization.

## Results

### Defining series of allelic mutants for conserved signaling genes and the *AeCRK* gene

To uncover the molecular mechanisms underpinning the Nod-independent symbiosis, an EMS-mutant-based approach was recently conducted in *A. evenia*. It led to the identification, in isolated nodulation mutants, of causal mutations for five genes of the Nod signaling pathway and the discovery of a symbiotic gene, *AeCRK* (Supplemental Table S1; Quilbé et al., 2021). To identify additional mutant alleles or genes, we conducted a mapping-by-sequencing approach on yet uncharacterized nodulation mutants. This approach applied to the mutant A21 by sequencing pooled DNAs from 140 F_2_ mutant plants, originating from a wild-type (WT) × A21 crossing, was successful in detecting a genetic linkage on chromosome Ae07 with a mutant allelic frequency reaching 98% at the *AeNIN* locus (Supplemental Figure S1). Another mutant, E26, was previously identified by a Targeted Sequence Capture as also being a candidate *nin* mutant, but was not genetically characterized further (Quilbé et al., 2021). We show that the mutant E26 has a monogenic and recessive determinism for its symbiotic phenotype, and the genotyping of 20 F_2_ backcrossed E26 mutant plants showed a strict co-segregation of the mutation with the symbiotic phenotype (Supplemental Table S2). Allelism tests with the A21 and E26 mutants, and the previously validated *nin* mutant, Y11, confirmed that they belong to the same complementation group (Supplemental Table S3). As a result, updated allelic mutant series are available for *AePOLLUX* (six alleles), *AeCCaMK* (four alleles), *AeCYCLOPS* (two alleles), *AeNSP2* (four alleles), *AeNIN* (seven alleles), and *AeCRK* (two alleles). They provide the opportunity for an in-depth comparative analysis because these genes can have different involvements and allelic mutations can have different effects on a gene due to complete or partial loss of function. For clarity, the corresponding mutants were renamed according to the gene name and the allele number (Supplemental Table S1).

### *nsp2* mutants lack nitrogen-controlled axillary root hairs normally colonized by bradyrhizobia

**Figure 1:**
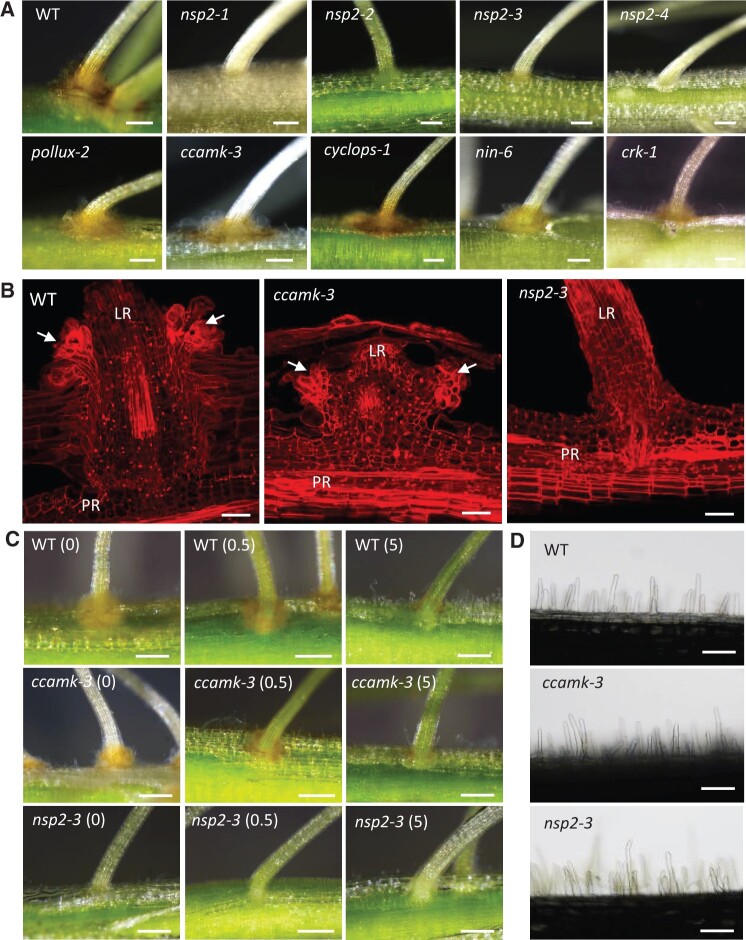
Absence of axillary root hairs in *nsp2* mutants of *A. evenia*. A, Axillary root hair outgrowth visible at the base of lateral roots of the WT plant and representative mutants of *AePollux*, *AeCCamK*, *AeCYCLOPS*, *AeNIN*, and *AeCRK* but not in the four allelic mutants of *AeNSP2.* B, Confocal analysis of lateral root bases in the WT line, and the ccamk-3 and nsp2-3 mutants. Cell walls were stained with Calcofluor White. Arrows point to axillary root hairs. LR: lateral root, PR: primary root. C, Effect of nitrogen on the growth of axillary root hairs in the WT line, and the *ccamk-*3 and *nsp2-3* mutants cultured in BNM medium complemented with a range of KNO_3_ concentrations (0, 0.5, and 5 mM). D, Root hair along the primary root in the WT plant, and the *ccamk-*3 and *nsp2-3* mutants. Bars = 250 µm (A and C), 50 µm (B), and 120 µm (D).

While all the *A. evenia* mutants were primarily screened for defect in nodulation, an unexpected observation was that all four *nsp2* mutants were completely devoid of axillary root hair crowns that normally surround lateral root bases (Figure 1A). These axillary root hairs are swollen structures covered with a thick mucilage and forming an orange-colored tuft. When present, bradyrhizobia intensively colonize their surface prior to inner root infection (Arrighi et al., 2012). In contrast to *nsp2* mutants, lines altered in other genes of the Nod signaling pathway, *AePOLLUX*, *AeCCaMK*, *AeCYCLOPS*, *AeNIN*, as well as *AeCRK*, have a normal development of axillary root hairs (Figure 1A). A closer examination of calcofluor-stained sections of lateral root bases by confocal microscopy showed similar tufts of thick-walled axillary root hairs in the WT and the *ccamk-3* mutant while in the *nsp2-3* mutant the lateral root bases showed a continuum root epidermis and no bulge-like structures were visible (Figure 1B).

Since nitrogen is known to negatively regulate the rhizobial symbiosis and axillary root hairs constitute the initial infection sites, we analyzed the potential effect of nitrogen on their development by supplying different KNO_3_ concentrations to the plant growth medium. Observations at 10-day postgermination revealed that, for the WT line and the *ccamk* mutant, axillary root hair crowns were easily visible with a treatment of 0- or 0.5-mM KNO_3_. However, a clear reduction of axillary root hair development was apparent upon the addition of 5-mM KNO_3_ (Figure 1C). In that case, only a discrete orange coloration was observable at the base of lateral roots. In contrast, no nitrogen effect was observed on the *nsp2* mutant, this latter is devoid of axillary root hairs (Figure 1C). We further explored the unusual phenotype observed in the *nsp2* mutants by assessing root hair development and the root system architecture. The four *nsp2* mutants produced root hairs with a similar tubular shape and with the same length as observed in WT plants and the *ccamk-3* mutant globally (Figure 1D;Supplemental Figure S2A). In contrast, significant variations in the primary root length, lateral root length, and number were found relative to the WT line (Supplemental Figure S2, B–D). However, these variations were opposed among the four *nsp2* mutants, suggesting that these variations are mutant-dependent rather than linked to *AeNSP2*. These observations reveal two features on the axillary root hairs present in *A. evenia*: (1) *AeNPS2* is important for their development and (2) nitrate has a negative effect on their formation.

### *nsp2* mutants show reduced expression of nitrogen-dependent genes

The potential link between *AeNSP2* and nitrogen homeostasis is supported by data from the *A. evenia* Gene Atlas that show that *AeNSP2* is expressed in roots but that this expression is repressed by the addition of nitrogen and decreases during nodulation (Quilbé et al., 2021). To investigate this singular expression pattern, we searched for genes co-expressed with *AeNSP2* and identified approximately 60 genes with a similar expression pattern. After an analysis of their putative biological functions, we retained a list of nine candidates with a potential role in symbiosis, root hair growth, or nitrogen homeostasis (Supplemental Table S4). In addition to *AeNSP2*, two other genes that we named as *AeERN1* and *AeERN3* encode predicted ERF required for nodulation (ERN) transcription factors, and they are the putative orthologs of the symbiotic genes *MtERN1* and *MtERN3* in *M. truncatula* (Andriankaja et al., 2007; Cerri et al., 2012). *AeCDD7* encodes a putative carotenoid cleavage dioxygenase 7 and likely corresponds to *MtCCD7* in *M. truncatula*, a gene previously shown to be involved in strigolactone biosynthesis and in rhizobium symbiosis (van Zeijl et al., 2015). *AeAMT3* is predicted to encode an ammonium transporter, while *AeHHP* encodes a putative histidine phosphotransfer protein involved in cytokinin signaling. Two genes may be involved in cell wall modifications, *AeEXP* (*expansin*) and *AePL* (*pectate lyase*). Lastly, *root hair defective 6-like 2* is predicted to encode a bHLH transcription factor that best matches with *AtRSL2* and *AtRSL4*, both required for root hair growth in *Arabidopsis thaliana*.

**Figure 2:**
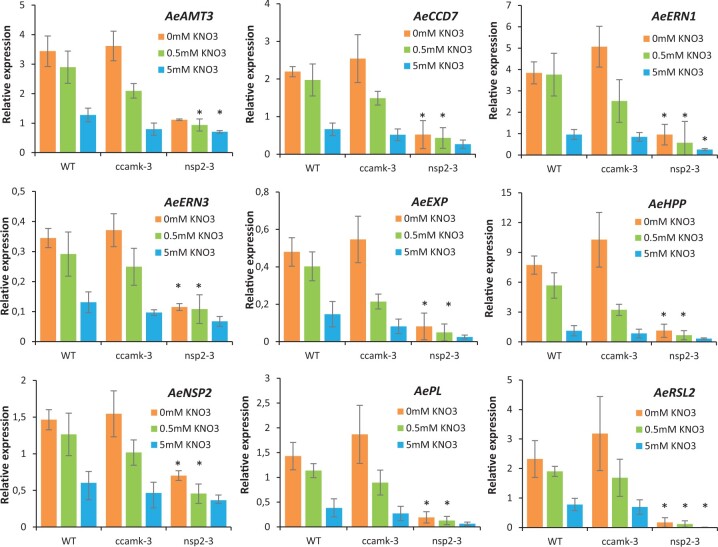
A role for *AeNSP2* in the expression of genes regulated by nitrogen in *A. evenia.* RT-qPCR analysis of *AeAMT3, AeCCD7*, *AeERN1*, *AeERN3*, *AeEXP*, *AeHPP*, *AeNSP*2, *AePL*, and *AeRLS2* expression in the WT plant and the *ccamk-3* and *nsp2-3* mutants cultured with a range of KNO_3_ concentrations (0, 0.5, and 5 mM) during 10 days. Expression values were normalized to *AeEF1a* and *Ubiquitin* levels. Means and sd were derived from three biological replicates, and asterisks indicate significant differences (*P < 0.05, Student’s *t* test) between mutants and WT for the same nitrogen treatment.

To experimentally verify that the nonsymbiotic expression of these genes is regulated by nitrogen and to test their dependency on *AeNSP2*, we assessed their expression by reverse transcription–quantitative PCR (RT–qPCR) under different nitrogen conditions (0-, 0.5-, and 5-mM KNO_3_) and in three genetic backgrounds (WT, *ccamK-3*, and *nsp2-3* mutants). In WT roots, expression of all nine genes was maximal in the absence of nitrogen (0-mM KNO_3_) and expression levels consistently decreased with increasing amounts of nitrogen. This negative relationship indicates that nitrogen has a repressive effect on the expression of these genes. A similar expression pattern was observed in the *ccamk-3* mutant roots, indicating that *AeCCaMK* does not participate in the repression of these genes (Figure 2). In contrast, under the same nitrogen treatments, gene expressions in the *nsp2-3* mutant roots of all tested genes were always lower than that in the WT line, and statistically significant for the 0- and 0.5-mM KNO_3_ conditions (Figure 2). These results suggest that *AeNSP2* is implicated, directly or indirectly, in the activation of expression of the nine identified genes.

### The symbiotic mutants show blocks at several steps of nodule development

**Figure 3:**
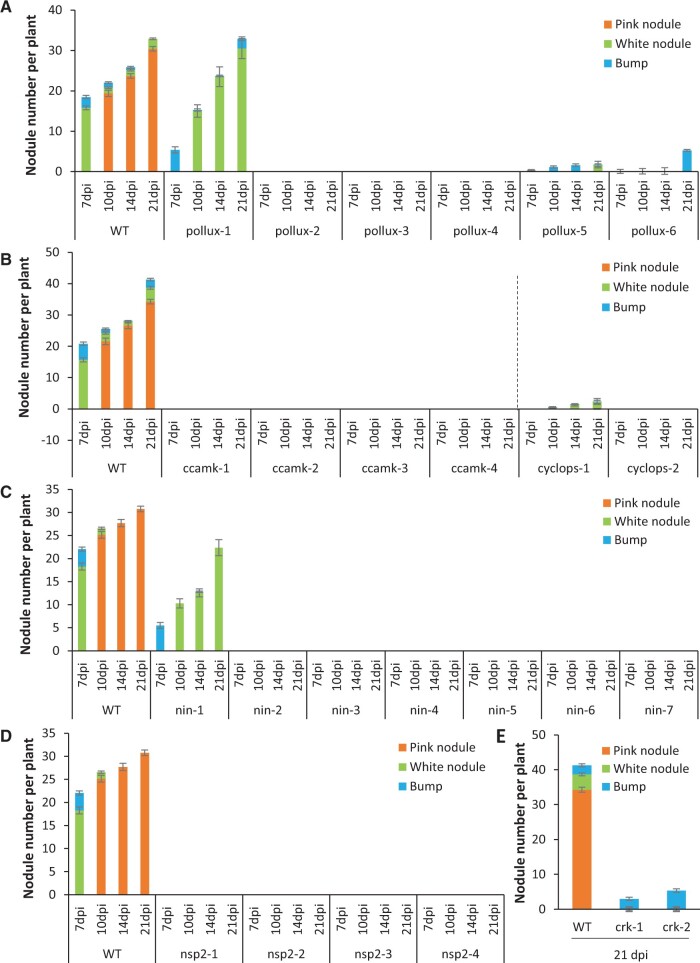
Nodulation kinetics of the nodulation mutants of *A. evenia* inoculated with *Bradyrhizobium* ORS278 and comparison with the WT line. A, *pollux* mutants. B, *ccamk* and *cyclops* mutants. C, *nin* mutants. D, *nsp2* mutants. E, *crk* mutants. Observations were performed at 4, 7, 10, and 14  dpi. Means and sd were derived from the phenotyping of 20 plants/line.

Next, we investigated the involvement of *AePOLLUX*, *AeCCaMK*, *AeCYCLOPS*, *AeNSP2*, *AeNIN*, and *AeCRK* in rhizobial symbiosis. For this purpose, we analyzed the nodulation kinetics of the six allelic mutant series after inoculation with *Bradyrhizobium* strain ORS278 over a time frame of 21 days. In all experiments, the WT line readily exhibited dark pink nodules upon inoculation and had well-developed green leaves. In contrast, all inoculated nodulation mutants showed symptoms of nitrogen starvation, having yellowish leaves, and a stunted growth habit at the end of the experiments (Supplemental Figures S3–S8). In the *pollux* series, *pollux-*2, *pollux-3*, and *pollux-4* mutants did not form nodules after inoculation. However, *pollux-6* produced a few bumps, and *pollux-1* and *pollux-5* formed nodules, albeit with consistently different frequencies (Figure 3A). For the four mutants of the *ccamk* series, the plants did not form nodule structures after inoculation while, in the *cyclops* series, the two mutants displayed different phenotypes, *cyclops-2* being devoid of any nodule-like structure and *cyclops-1* developing nodules at low frequency (Figure 3B). In the *nin* series, most mutants were completely nonnodulating; however, *nin-1* had a less severe phenotype since it was able to develop nodules (Figure 3C). In the *nsp2* series, the phenotype was found to be homogeneous, with all mutants having noduleless roots (Figure 3D). Finally, in the *crk* series, the two mutants displayed an apparent null phenotype; however, microscopic examination at 21-day postinoculation (dpi) revealed the presence of a few very small bumps on their roots (Figure 3E). For nodule-producing mutants, the nodules tended to be smaller and paler than those of the WT plants, and acetylene reduction assays confirmed that the nodules had no (*nin-1*) or a weak (*pollux-1*, *pollux-5*, and *cyclops-1*) nitrogenase enzyme activity (Supplemental Figures S3, S5, and S7).

The obtained data show that three main symbiotic phenotypes can be distinguished within the isolated nodulation mutants: Nod^−^ (no nodule at all), Bump^+^ (limited nodule development), and Fix^−^ (ineffective nodules). Intriguingly, while the Nod^−^ phenotype was found in all allelic series for the conserved signaling genes, the two *crk* mutants have a similar Bump^+^ phenotype. Therefore, we questioned the impact of these mutations on AeCRK function. First, to assess the predicted altered splicing in the *crk-1* mutant, we amplified *AeCRK* cDNAs in the WT and the *crk-1* mutant backgrounds (Supplemental Figure S9). While a single band of the expected size was readily obtained for the WT, three bands were recovered for *crk-1*. Their sequencing revealed variations that are predicted to lead to abnormal forms of AeCRK. Second, *crk-2* has a G354E mutation falling in the first Gly of the highly conserved Gly-rich loop (GXGXXG), which is involved in ATP binding and essential for kinase activity (Quilbé et al., 2021). This observation prompted us to assay the kinase autophosphorylation activity, using anti-phospho-Thr antibodies, of the WT and mutated (G354E) forms of the AeCRK kinase domain produced in *Escherichia coli* and purified as GST-tagged proteins. In contrast to the two dead-kinase proteins used as control, GST-CRKkin-G354E phosphorylation was detectable, but was about 80-fold less than for the WT AeCRK kinase protein (Supplemental Figure S10). These results show that AeCRK has an active kinase domain and that the G354E mutated form present in the *crk-2* mutant retains only residual kinase activity. Therefore, both *crk-1* and *crk-2* mutations can be considered as having a strong impact on AeCRK function.

### The symbiotic mutants are differently altered in rhizobial infection

To analyze further the nodulation phenotypes evidenced with the *Bradyrhizobium* ORS278 strain, we focused within each allelic mutant series on mutants that had a clear difference in symbiotic phenotype. First, we determined whether these plant phenotypes were dependent on the bradyrhizobial strain used for inoculation. For this, the symbiotic phenotype of selected mutant lines at 21 dpi with *Bradyrhizobium* strain ORS285, which is also compatible but differs in its infectivity, was determined (Supplemental Figures S11–S16). Subsequently, we analyzed for these mutants the bacterial infection and nodule development using GUS-tagged versions of either ORS278 or ORS285.

**Figure 4:**
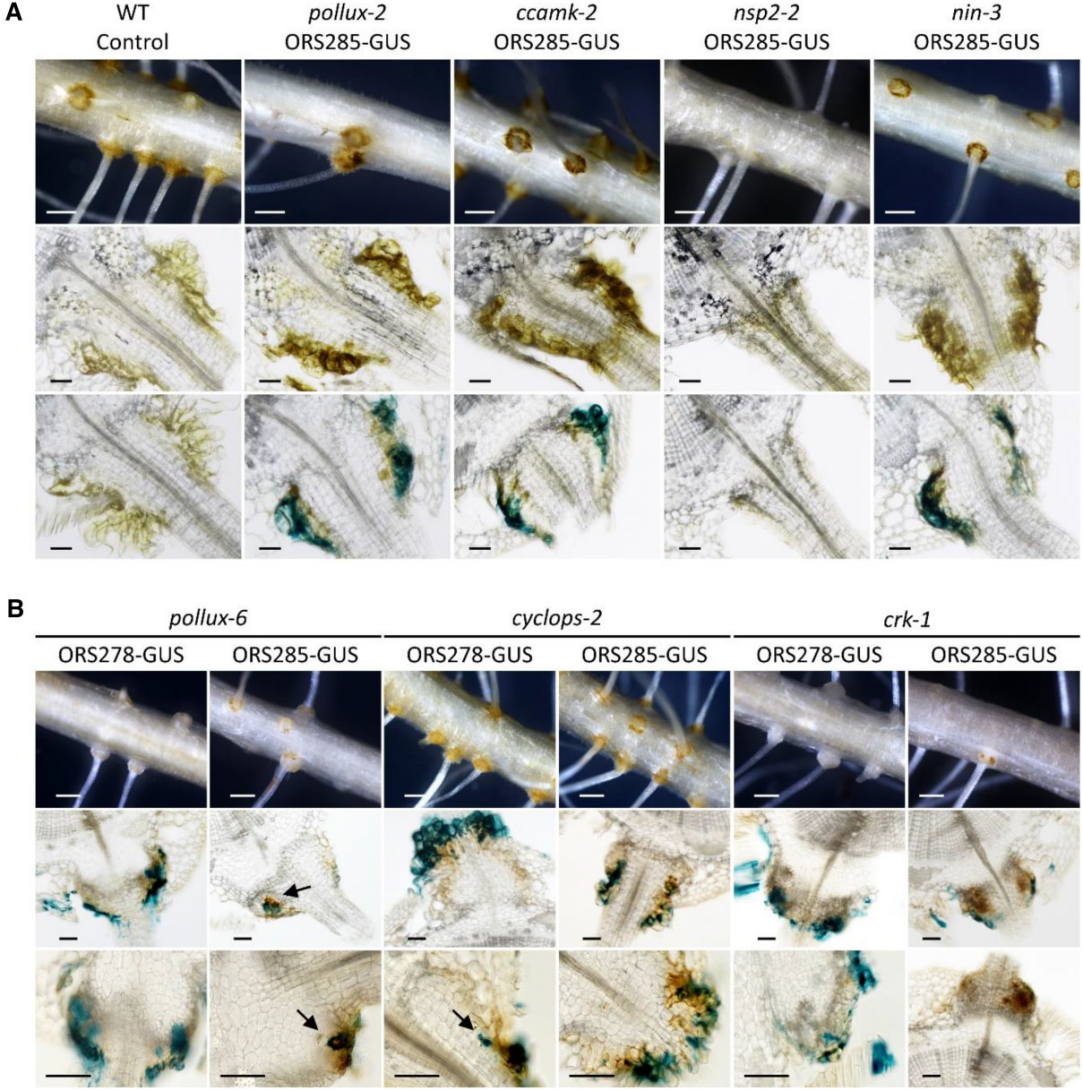
Bradyrhizobial infection in Nod^−^ and Bump^+^ mutants. A, Mutant lines showing only bacterial colonization of the axillary root hairs. B, Mutants showing inner root infection. Whole roots (upper) and 70-µm-thick root sections before and/or after X-Gluc staining (middle and lower) of control WT (uninoculated) and mutant lines inoculated with either the ORS278-GUS or ORS285-GUS strain. Roots were harvested at 21 dpi. Arrows indicate inner infection spots. Bars represent 500 µm (upper) or 50 µm (middle and lower).

The results show that plants from the mutant lines, *pollux-2*, *ccamk-2*, *cyclops-2*, *nsp2-2*, and *nin-3*, had also a Nod^−^ phenotype when inoculated with the ORS285 strain. Macro and microscopic analysis of whole and sectioned roots evidenced again the absence of axillary root hairs in the *nsp2-2* mutant, and no bacteria were detected on the root surface of this mutant after X-Gluc staining (Figure 4A). In contrast, the remaining mutants developed orange axillary root hair rosettes. They tended to be darker on roots from inoculated plants than on noninoculated roots, and X-Gluc staining revealed that they were colonized by bradyrhizobia (Figure 4A). A closer examination revealed that on *pollux-2*, *ccamk-2*, and *nin-3* mutants, bacterial colonization was restricted to or in between axillary root hairs. The *cyclops-2* mutant differed by displaying either a few inner infection spots or brownish areas, when inoculated with the strain ORS278-GUS or ORS285-GUS, respectively (Figure 4B). The mutant lines *pollux-6* and *crk-1*, that developed bumps with the ORS278 strain, formed more discrete and infrequent bumps when inoculated with the ORS285 strain. Unlike the typical WT lobe-shaped bumps, these bumps appeared to be circular swellings, suggesting homogeneous but limited cell divisions all around the base of the lateral roots (Figure 4B). In sectioned mutant bumps, X-Gluc-stained ORS278 bacteria were contained in infection pockets, suggesting early aborted infection (Figure 4B). When the *pollux-6* and *crk-1* mutants were inoculated with the ORS285-GUS strain, the axillary root hair crowns were noticeably characterized by punctate darker zones, linked with underlying bacterial infection spots and brownish coloration that might correspond to defense reactions (Figure 4B). Thus, although ORS285 induced less cell divisions than ORS278, a similar premature aborted infection was observed.

**Figure 5:**
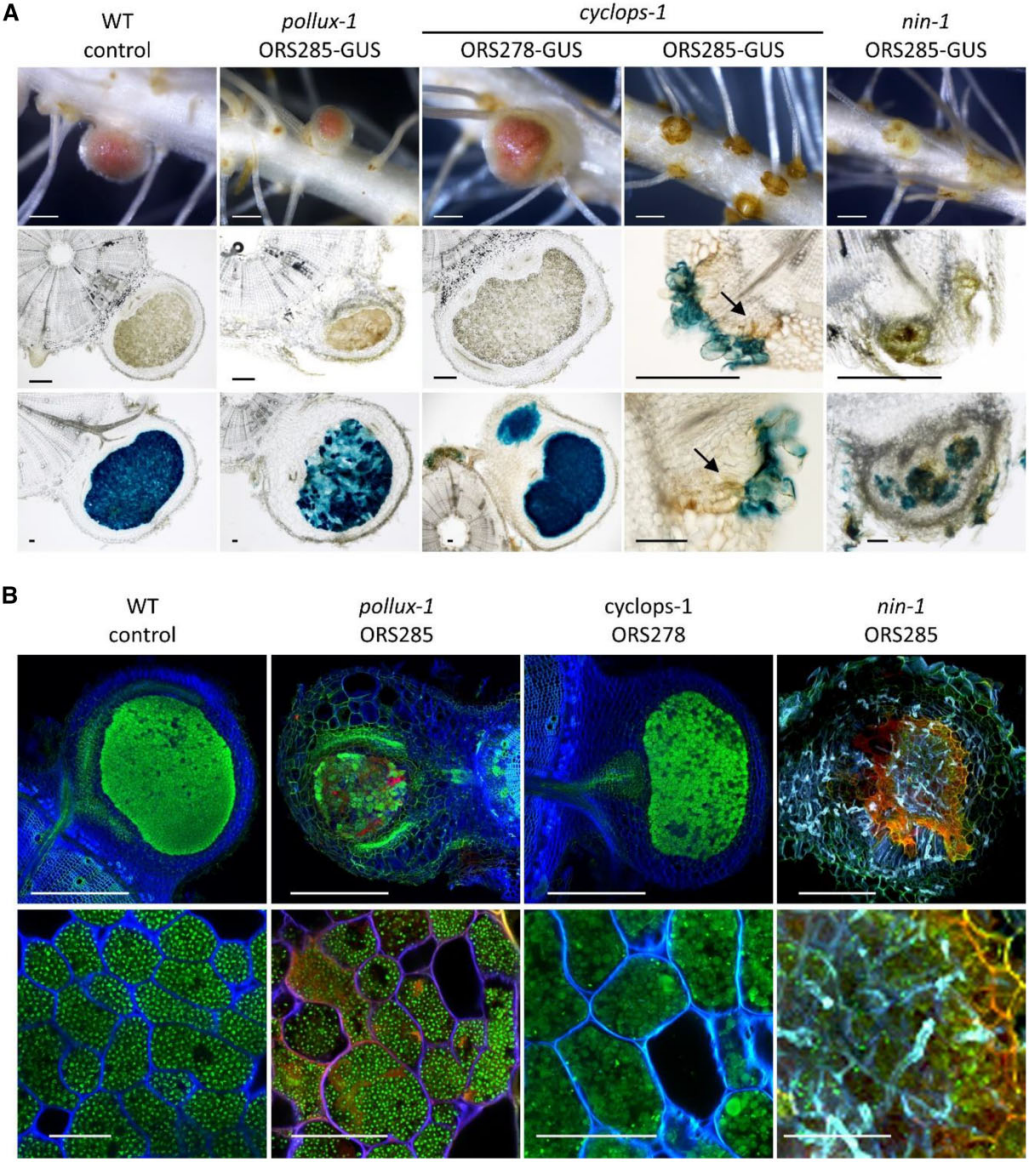
Bradyrhizobial infection and differentiation in Fix^−^ mutants. A, Nodule infection. Whole roots (upper) and 70 µm-thick root or nodule sections before and after X-Gluc staining (middle and lower, respectively) of WT) and mutant lines inoculated with either the ORS278-GUS or ORS285-GUS strains. Roots were harvested at 21 dpi. Arrows indicate inner infection spots. B, Bacterial differentiation. Confocal microscopy observation of longitudinal 70 µm-thick sections of nodules 21 dpi with the ORS278 or ORS285 strain stained with SYTO13 (upper) and magnification of infected nodule cells to show bacterial morphology. Bars in (A) represent 500 µm (upper), 250 µm (middle), and 50 µm (lower). Bars in (B) represent 200 µm (upper) and 50 µm (lower).

Among the three mutants forming Fix^−^ nodules with ORS278 (*pollux-1*, *nin-1* and *cyclops-1*) only the *cyclops-1* mutant was Nod^−^ after inoculation with the strain ORS285 (Figure 5A). The *pollux-1* mutant developed two types of nodules with the ORS285 strain. The majority of the nodules was small, round in shape, and contained a brownish spot while a small number of nodules were better developed and had a pink color. However, infection of the central tissue was noticeably patchy (Figure 5A). In contrast, whereas ORS278-induced nodules on the *cyclops-1* mutant had a pink color, they were homogeneously infected, similarly to WT plants (Figure 5A). Finally, after inoculation with ORS285, the *nin-1* mutant had the most severe phenotype, forming only white small rounded nodules. Sections of these nodules showed a limited bradyrhizobial infection and the presence of brown phenolic compounds (Figure 5A). To investigate the bacterial differentiation in nodules of the Fix^−^ mutants, we used the nucleic acid-binding dye SYTO13 and observed the bacterial morphology by confocal microscopy (Figure 5B). Mature nodules of the WT plants contained typical spherical bacteroids and a similar morphology was observed for bacteria in the nodules of the *pollux-1* mutant, suggesting that they are differentiated. In contrast, nodules of the *cyclops-1* mutant contained bacteria with abnormal shape and size, showing that the process of bacteroid differentiation was altered. Finally, nodules of the *nin-1* mutant contained only elongated bacteria, pointing to a failure of bacterial differentiation. These also showed a strong autofluoresence that is related to the brown pigmentation described above (Figure 5A). These confocal observations of bacterial infection and differentiation were in line correlated with the very low nitrogenase enzyme activity of *pollux-1* and *cyclops-1* nodules and its complete absence in *nin-1* nodules with the ORS278 or ORS285 strain (Supplemental Figures S3, S5, S7, S11, S13, and S15).

### The symbiotic genes are required for normal expression of early nodulin genes

Nodule organogenesis and bacterial infection are accompanied with the induction of numerous genes qualified as nodulins. To examine the effect of mutations in symbiotic genes on nodulin transcription, we selected a set of six genes whose expression is induced during nodulation, according to the *A. evenia* Gene Atlas (Supplemental Table S4) (Quilbé et al., 2021). Four of them—*AeNIN*, s*ymbiotic remorin (AeSymREM1)*, *vapyrin (AeVPY)*, and *AeENOD40—*are putative orthologs of genes with a well-described symbiotic function in model legumes. *NIN* is a central regulator of nodulation (Liu et al., 2019). *SymREM1* and *VPY* are important for rhizobial infection (Lefebvre et al., 2010; Murray et al., 2011). *Early nodulin 40 (ENOD40*) is required for the induction of cortical cell divisions (Charon et al., 1999). The two remaining nodulin genes, *AeSBT* (*subtilase*) and *AeCRK*, were selected based on transcriptomic and genetic studies on *A. evenia* symbiosis (Quilbé et al., 2021; Gully et al., 2018).

**Figure 6:**
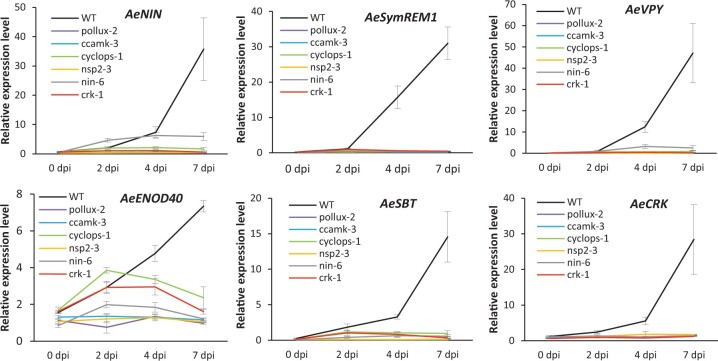
Expression of early nodulation genes in the WT and the mutants *pollux*, *ccamk*, *cyclops*, *nsp2*, *nin*, and *crk* in *A. evenia*. Expression of *AeNIN, AeSymREM1*, *AeVPY*, *AeENOD40*, *AeSBT*, *AeNSP*2, and *AeCRK* was determined during nodulation kinetics with *Bradyrhizobium* ORS278 at 0, 2, 4, and 7  by RT–qPCR analysis. Expression values were normalized to *AeEF1a* and *Ubiquitin* levels. Means and sd were derived from three biological replicates.

We monitored the expression of these rhizobia-induced genes on mutants of *AePOLLUX*, *AeCCaMK*, *AeCYCLOPS*, *AeNSP2*, *AeNIN*, and *AeCRK*, and the WT line over a period of 7 days following inoculation with *Bradyrhizobium* strain ORS278. RT–qPCR showed a clear induction of the analyzed nodulin genes at 2 or 4 dpi and still increasing at 7 dpi in the WT (Figure 6). In contrast, induction of expression of *AeSymREM1*, *AeVPY*, *AeSBT*, and *AeCRK* was totally abolished in all six tested mutant lines (Figure 6). For *AeNIN*, induction of expression was severely reduced at 7 dpi in the *nin-6* mutant background, and this induction was completely impaired in the other symbiotic mutants (Figure 6). For *AeENOD40*, its level of expression remained unchanged after inoculation in the *pollux-2*, *ccamk-3*, *nsp2-3*, and *nin-6* mutants, whereas a low and transitory induction was visible for the *cyclops-1* and *crk-1* mutants. The latter is consistent with the absence of a null phenotype as observed for these mutants (Figure 6). The above results indicate that, as described in model legumes, nodulin gene expression is dependent on the symbiotic signaling pathway in *A. evenia* and that *AeCRK* is also required to assure normal symbiotic induction of the tested nodulin genes.

### Certain nodulation mutants are defective for arbuscular mycorrhizal symbiosis

**Figure 7:**
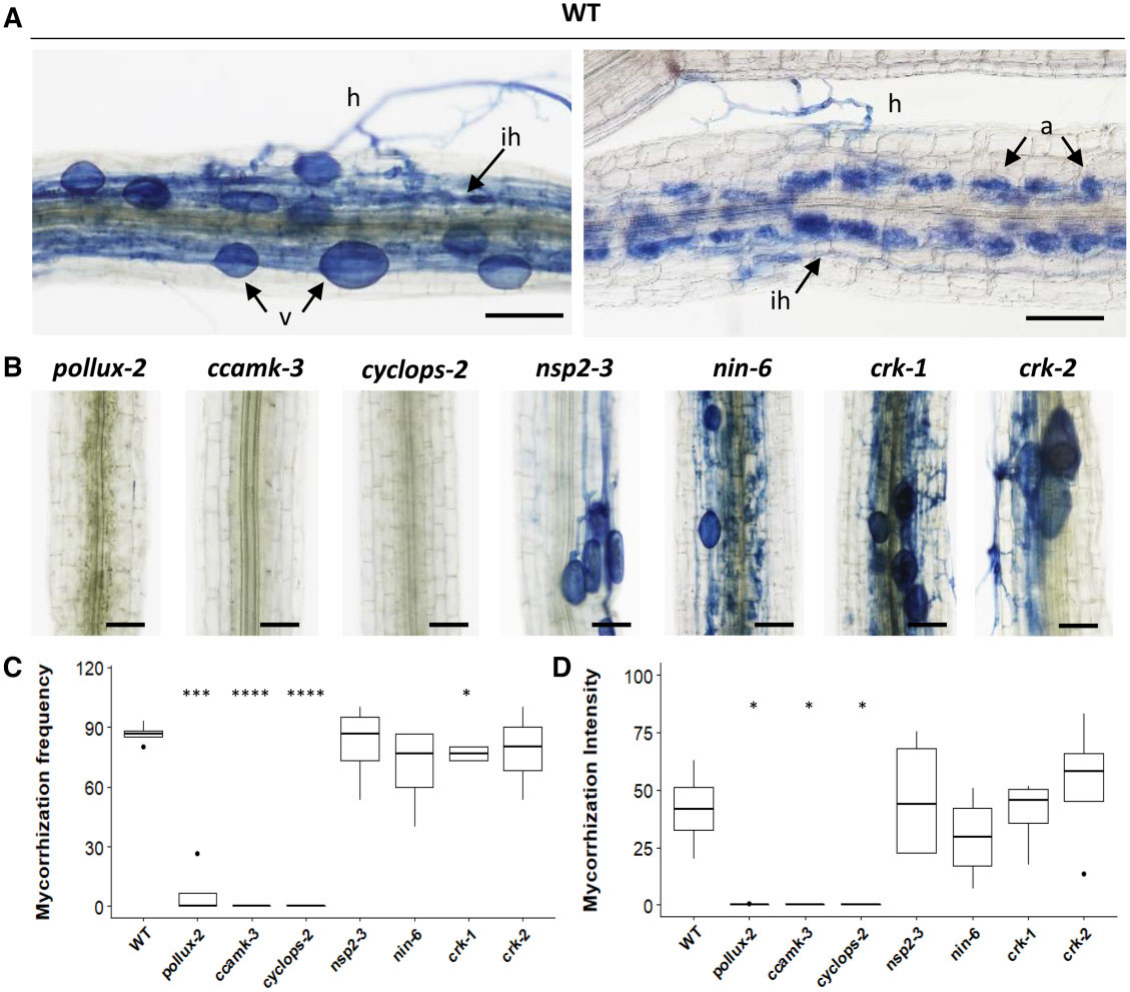
Mycorrhizal phenotypes of the WT and the mutants *pollux*, *ccamk*, *cyclops*, *nsp2*, *nin*, and *crk* in *A. evenia*. Plant roots of the different lines cultivated for 8 weeks and inoculated with *R. irregularis* were stained with ink for microscopy analysis and quantification of fungal colonization according to the Mycocalc method. A, Typical fungal structures observed in the WT roots with hyphae (h), intraradical hyphae (ih), arbuscules (a), and vesicles (v). B, Representative fungal colonization patterns observed in the different mutant lines. C and D, Box plots represent the mycorrhization frequency and intensity, respectively, both expressed in percentage in the WT and mutant lines. The central rectangle spans the first quartile to the third quartile; the bold segment inside the rectangle shows the median; and the whiskers above and below the box show the locations of the maximum and minimum value, respectively. Outliers are represented by dots. Data are from three biological repeats, each with five plants/line. Statistical analyses were performed using Student’s *t* test (*P < 0.05, ***P < 0.001, ****P < 0.0001) by comparing the mutant lines to the WT. Bars = 100 µm (A) and 50 µm (B).

In model legumes, many genes of the Nod signaling pathway or involved in rhizobial infection have been shown to play a role in arbuscular mycorrhizal symbiosis. However, in the view of the semi-aquatic habit of *A. evenia*, an environment unfavorable for mycorrhizal symbiosis, and the absence of literature data, it is not known if this species is able to establish such a fungal symbiosis. Therefore, we first assessed the ability of *A. evenia* WT to interact symbiotically with *Rhizophagus irregularis* under aeroponic conditions. Eight weeks after inoculation, typical fungal structures could be observed on the roots (Figure 7A). The presence of both extra- and intra-cellular hyphae was indicative of effective root infection. In addition, inner cortical cells with well-developed arbuscules and vesicles suggested that this symbiosis is fully developed. Next, we questioned whether *AePOLLUX*, *AeCCaMK*, *AeCYCLOPS*, *AeNSP2*, *AeNIN*, or *AeCRK* are important for mycorrhization in *A. evenia*, by evaluating the mycorrhizal phenotype of one strong allele mutant for each signaling gene and the two allelic mutants available for *AeCRK*. Microscopic analysis of the fungi-inoculated roots revealed that the *nsp2-3*, *nin-6*, *crk-1*, and *crk-2* mutants are mycorrhized, whereas the *pollux-2*, *ccamk-3*, and *cyclops-2* mutants showed almost no mycorrhizal colonization (Figure 7B). When fungal colonization was quantified as the mycorrhization frequency, there was no difference between the *nsp2-3*, *nin-6*, and *crk-2* mutants and the WT line (Figure 7C). Although the mycorrhization intensity was more variable between the *nsp2-3*, *nin-6*, *crk-1*, and *crk-2* mutants and the WT line, these variations were not significantly different (Figure 7D). Thus, these phenotypic observations support a major role of *AePOLLUX*, *AeCCaMK*, and *AeCYCLOPS* for the mycorrhizal symbiosis in *A. evenia*.

**Figure 8:**
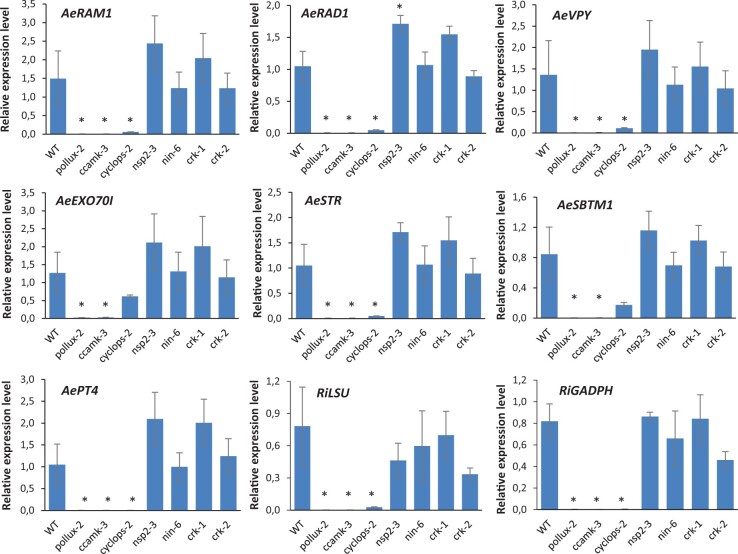
Expression of mycorrhization-induced genes in the WT and the mutants *pollux*, *ccamk*, *cyclops*, *nsp2*, *nin*, and *crk* in *A. evenia.* Expression of *AeRAM1*, *AeRAD1*, *AeVPY*, *AeEXO70I*, *AeSTR*, *AeSBTM1*, *AePT4*, and of the two fungal genes *RiLSU* and *RiGADPH* was determined by RT–qPCR analysis on plant roots cultivated for 8 weeks and inoculated with *R. irregularis*. Expression values were normalized to *AeEF1a* and *Ubiquitin* levels. Means and sd were derived from three biological replicates. Asterisks indicate significant differences (^*^P < 0.05, Student’s *t* test) between mutants and WT.

To further characterize the mycorrhizal symbiosis in *A. evenia*, we developed seven plant mycorrhization markers by identifying putative orthologs of mycorrhiza-induced genes described in *M. truncatula* (Supplemental Table S5) (Takeda et al., 2011; Park et al., 2015; Gibelin-Viala et al., 2019). Among them, *reduced arbuscular mycorrhization 1 (AeRAM1*) and *required for arbuscule development (AeRAD1*) are involved in mycorrhizal signaling. *AeVPY*, *exocyst 70I (AeEXO70I*), *stunted arbuscule (AeSTR*), and *arbuscular mycorrhiza-induced subtilase 1 (AeSBTM1*) are markers for mycorrhizal infection and arbuscule development, while *phosphate transporter 4 (AePT4*) is involved in arbuscule functioning. In addition to plant markers, two mycorrhiza-specific genes, 25S-large ribosomal subunit (*RiLSU*) and *glyceraldehyde 3-phosphate dehydrogenase (RiGADPH*), were selected (Xue et al., 2015; Buendia et al., 2016). All seven plant genes were checked to be effectively induced during mycorrhization and the two mycorrhiza-specific genes to enable quantification of the fungal colonization of the roots (Supplemental Figure S17). Next, we measured the expression levels of these selected mycorrhizal marker genes by RT–qPCR in WT and mutant plant roots. Although variability could be found between the WT line and the mycorrhized *nsp2-3*, *nin-6*, *crk-1*, and *crk-2* mutants, in most cases, these differences were statistically not significant (Figure 8). In contrast, consistent with the observed absence of fungal colonization in the roots of the *pollux-2*, *ccamk-3*, and *cyclops-2* mutants, fungal genes were hardly detected, and plant marker genes were not induced or showed a very much reduced expression level (Figure 8).

**Figure 9:**
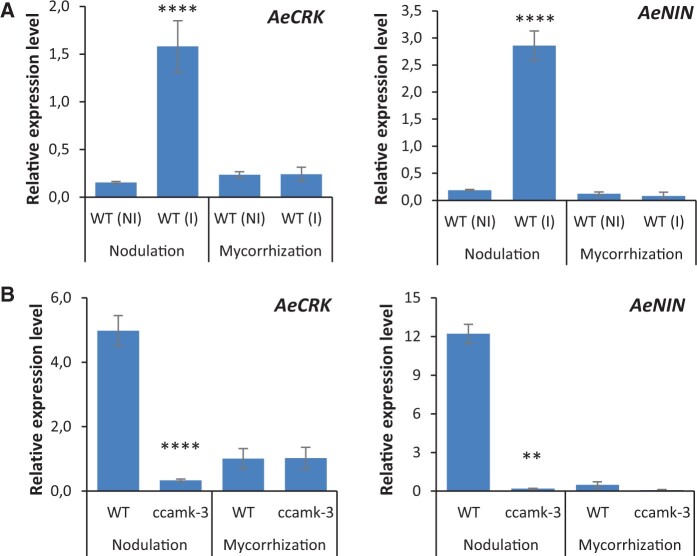
Comparative expression of *AeCRK* and *AeNIN* during nodulation and mycorrhization. A, Gene expression levels in the WT line either noninoculated (NI) or inoculated (I) with the symbiont. B, Gene expression in the WT and the *ccamk* mutant inoculated with the symbiont. Expression levels were determined at 7 dpi with *Bradyrhizobium* ORS278 and at 8-week postinoculation with *R. irregularis* by RT–qPCR analysis. Expression values were normalized to *AeEF1a* and *Ubiquitin* levels. Means and sd were derived from four (A) and seven (B) biological replicates. Asterisks indicate significant differences (^*^P < 0.05, Student’s *t* test) between the control and the other samples.

Exploiting the observation that both *AeCRK* and *AeNIN* genes are induced during nodulation, we investigated if such upregulation of expression also occurs during mycorrhization, as is the case for *AeVPY* (Figures 6 and 8). For this purpose, a comparative analysis of WT plant roots, inoculated with *Bradyrhizobium* ORS278 (nodulation) or *R. irregularis* (mycorrhization), was conducted by RT–qPCR. Concordant with other transcriptomic data, an increase in *AeCRK* and *AeNIN* expression was observed in nodulated roots relative to control roots (Figure 9A). In contrast, no significant difference in expression was detected between mycorrhized roots and noninoculated roots (Figure 9A). We also compared *AeCRK* and *AeNIN* expression during the two symbioses in the WT line and the completely symbiosis-deficient *ccamk-3* mutant. While in rhizobial symbiosis their expression levels were consistently higher in WT roots as compared to *ccamk-3* roots, no such differences were observed during mycorrhizal symbiosis (Figure 9B). Thus, the absence of both a mycorrhizal phenotype and the induction of symbiotic gene expression during symbiosis with *R. irregularis* suggest that *AeCRK* and *AeNIN* are not involved in mycorrhization in *A. evenia*.

## Discussion

**Figure 10:**
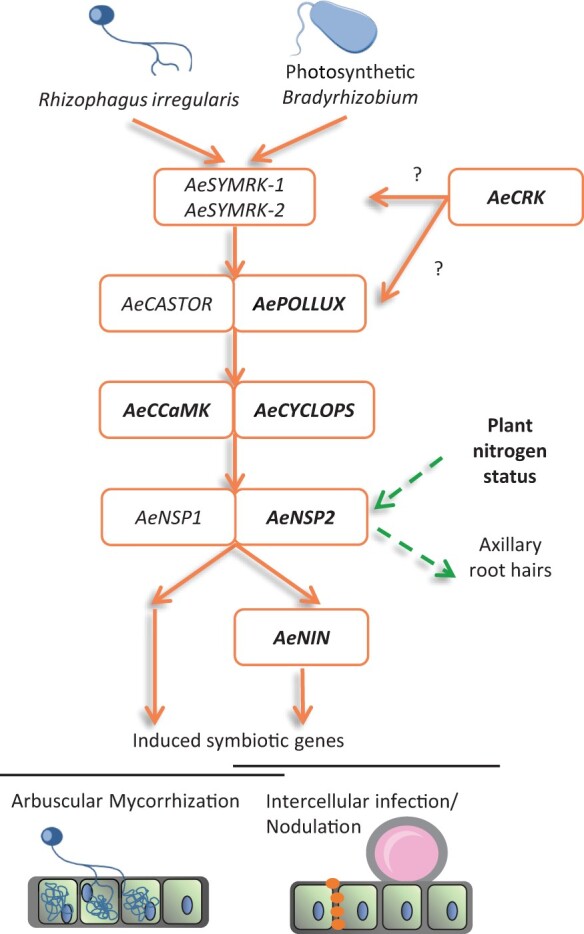
Schematic model depicting symbiotic gene involvements in *A. evenia*. Mutant-based identified genes are in bold, other genes are inferred from model legumes. The symbiotic signaling pathway and the recently identified gene *AeCRK* are required for Nod-independent signaling and intercellular infection. *AePOLLUX*, *AeCCaMK*, and *AeCYCLOPS* are also important for arbuscular mycorrhization. *AeNSP2* has as an additional nonsymbiotic function to regulate nitrogen-dependent responses including axillary root hair formation. ‘?’ represents how *AeCRK* connects to the symbiotic signaling pathway remains to be determined. This representation is adapted from Quilbé and Arrighi (2021).

In this study, we performed a phenotypic and molecular analysis on a series of allelic mutants for the genes *AePOLLUX*, *AeCCaMK*, *AeCYCLOPS*, *AeNIN*, and *AeCRK*. This allowed us to specify the modalities of the establishment of the Nod-independent symbiosis in *A. evenia* and it provided insights into the biological functions that these genes fulfill (Figure 10). Here, we discuss three main features of these gene involvements in *A. evenia* and how this information contributes to a better understanding of the mechanisms underpinning the establishment of symbioses in legumes.

### Involvement of the symbiotic signaling pathway in intercellular rhizobial infection and mycorrhization

Knowledge acquired on rhizobial and mycorrhizal symbioses in model legumes facilitates research in other legume species that have alternative symbiotic processes. So far, variations to the major studied nodulation mechanisms have been best investigated in two robinoid legumes, *Sesbania rostrata* and *L. japonicus*, which exhibit dual rhizobial infection pathways (partially intercellular infection and infection thread formation) (Capoen et al., 2010; Montiel et al., 2021). The Nod signaling pathway was recently shown to be equally important for both infection routes in *L. japonicus* (Montiel et al., 2021). *Arachis hypogaea* (peanut) and *A. evenia*, belonging to the Dalbergioid clade, are two examples of legumes that only use an intercellular infection process. Genetic studies showed that several genes of the Nod signaling pathway are conserved and important for symbiosis in both these legumes (Fabre et al., 2015; Sharma et al. 2020; Quilbé et al., 2021; Peng et al., 2021 ). Here, we specified the involvement of symbiotic signaling genes of *A. evenia* in intercellular infection. We showed that strong mutants for the *AePOLLUX*, *AeCCaMK*, *AeNSP2*, and *AeNIN* genes were characterized by a Nod^−^ phenotype, where the bradyrhizobia remain at the surface of axillary root hairs. Lack of bacterial penetration resembles the phenotype repeatedly described for mutants of the Nod signaling pathway in model legumes and indicates that this pathway also intervenes in the initial step of the symbiotic interaction in *A. evenia*. Besides a complete Nod^−^ phenotype, we evidenced among the allelic series of mutants of *AePOLLUX*, *AeCYCLOPS*, and *AeNIN* genes a spectrum of nodulation phenotypes. The broadest gradation was obtained for *AePOLLUX*, with the *pollux-1* and *pollux-6* mutants that are classified as Bump^+^ or Fix^−^. In these mutants, infection was impaired with abortive infection pockets in mutant bumps and scattered infection present in mutant nodules. A somewhat similar picture was obtained for *AeNIN*, for which the *nin-1* mutant is able to develop nodules but with a severely impaired bacterial infection and differentiation. This phenotyped the few weak *nin* phenotypes observed in *M. truncatula* and supported the recent evidence that *NIN* is important not only for nodule inception but also for transition to nitrogen fixation (Liu et al., 2021; Feng et al., 2021). Interestingly, both mutants for *AeCYCLOPS* were completely Nod^−^ with *Bradyrhizobium* strain ORS285, while two distinct phenotypes were observed with *Bradyrhizobium* strain ORS278. The strong phenotype, found in *cyclops-2*, equated to infection pockets that were not associated with bump formation and the weaker phenotype, observed in *cyclops-1*, corresponded to a few nodules that contained bacteria altered in their accommodation. This situation is reminiscent of the ones described for the *ipd3-2*/*ipd3l* double mutant and the *ipd3-2* mutant of *M. truncatula*, respectively (Horváth et al., 2011; Jin et al., 2018). All these observations highlighted, despite the important differences between intercellular and intracellular infections, a similar involvement of the Nod signaling pathway all throughout the process of rhizobial invasion and symbiosome formation (Figure 10). *Aeschynomene evenia* also provided a good opportunity to assess an involvement in mycorrhiza formation of symbiotic signaling genes. Mutant phenotyping indicated that mutations in *AePOLLUX*, *AeCCaMK*, and *AeCYCLOPS* drastically affected root colonization *by R. irregularis*, in contrast to *AeNSP2* and *AeNIN*. In *M. truncatula*, *NSP2* was found to facilitate mycorrhizal root colonization, although it is not essential to trigger mycorrhizal symbiosis (Maillet et al., 2011). In contrast, *MtNIN* was demonstrated to be not required and not induced during mycorrhization (Kumar et al., 2021). We found that *AeNIN* was not induced either during mycorrhization*.* Therefore conserved signaling genes in *A. evenia* appear to be similarly involved or not in mycorrhization as in model legumes (Figure 10) (Gobbato, 2015; Kumar et al., 2021).

### *AeNSP2* has functions in symbiotic signaling and the control of rhizobia-colonized axillary root hair development

In the Nod signaling pathway, NSP2 is a key transcriptional regulator that regulates the expression of symbiotic genes (Quilbé and Arrighi, 2021). In *A. evenia*, phenotypic and molecular data obtained for the *nsp2* mutants suggest that this function is conserved (Figure 10). However, *AeNSP2* has a singular expression profile, being repressed in roots by nitrogen and during nodulation (Quilbé et al., 2021). This negative regulation was not restricted to *AeNSP2* and actually involved a small set of genes. We showed for nine of these genes that their expression was not only dependent on nitrogen but also on *AeNSP2*, suggesting that they are directly or indirectly activated by *AeNSP2*. A nonsymbiotic function for *NSP2* was previously reported in *M. truncatula* by Liu et al. (2011). In this legume, NSP2 is important for the expression of the *DWARF27* gene that intervenes in the carotenoid and strigolactone biosynthetic pathway. Here we show that in *A. evenia*, another gene of this biosynthetic pathway, *CCD7*, is expressed in roots in an *NSP2*-dependent manner. Therefore, it is likely no coincidence that the *DWARF27* and *CCD7* genes appear to be co-expressed during nodulation in *M. truncatula*, suggesting that they have been conjointly co-opted in rhizobium symbiosis (van Zeijl et al., 2015). Additionally, we show that *NSP2* is required for the expression of two genes of the symbiotic signaling pathway, *AeERN1* and *AeERN3*. In *M. truncatula*, ERN1 and ERN2 were reported to be involved in a complex interplay with NSP1 and NSP2 GRAS factors and that ERN3 represses ERN1/ERN2-dependent transcription activation (Andriankaja et al., 2007; Cerri et al., 2012). Most intriguingly was the discovery that *NSP2* and nitrogen are major determinants controlling axillary root hair formation in *A. evenia*. These axillary root hairs form rosettes at the base of lateral roots that correspond to bradyrhizobial infection sites in various legumes, such as *Aeschynomene spp.*, *S. rostrata*, and peanut (Lievens et al., 2005; Bonaldi et al., 2011; Peng et al., 2018). Our finding is consistent with the recent identification of natural sequence variations compromising *NSP2* function in peanut lines that are defective in both nodulation and axillary root hair formation (Peng et al., 2021, 2018). The three above examples illustrate that *NSP2* has a regulatory function that is tightly linked to the plant nutrient status (Figure 10). Although this regulation occurs in nonsymbiotic conditions, some *NSP2*-regulated genes or structures have important roles in symbioses, suggesting that its dual functions are connected as suggested by Quilbé and Arrighi (2021). *Aeschynomene evenia* offers opportunities for investigations of the nutrient-related function of NSP2, notably by using its axillary root hairs as morphological markers. In this context, it should be mentioned that axillary root hairs in *A. evenia* are distinct from those encountered in other legumes, by being thickly covered with a mucilage that bradyrhizobia intensely colonize prior to their progression into the root (Bonaldi et al., 2011; Arrighi et al., 2012). Elucidating the genetic control underpinning their ontogenesis and functioning in *A. evenia* could also solve the question of whether axillary root hairs represent an adaption to enable initiation of infection during the Nod-independent symbiosis.

### *AeCRK* is important for symbiotic signaling and early rhizobial infection but not mycorrhization

A distinct component used by *A. evenia* to trigger nodulation is the *AeCRK* gene that belongs to the large plant *CRK* gene family in which many members have been associated with reactive oxygen species (ROS) sensing (Quilbé et al., 2021; Bourdais et al., 2015). To assess *AeCRK* roles, we investigated its involvement in mycorrhiza formation. Mutant phenotyping indicated that mutations in *AeCRK* do not affect root colonization by *R. irregularis*. Furthermore, *AeCRK* was shown to be strongly induced during nodulation but not during mycorrhization*.* This analysis showed that, similar to *AeNIN*, *AeCRK* symbiotic function seems to be specific to nodulation (Figure 10) (Kumar et al., 2021). When inoculated with *Bradyrhizobium* strain ORS278, *crk* mutants developed swellings that circled lateral roots and which often contained an infection pocket. These circular bumps were reminiscent of those spontaneously induced in *A. evenia* lines expressing deregulated forms of *AeCCaMK* (Fabre et al., 2015). This suggests that, in *crk* mutants, cortical cell divisions occur to form a bump, but that a positional information of the bacteria is missing to form a nodule. The infection pockets located in the outer cortical cell layers indicated an early block in the infection process. Interestingly, in WT *A. evenia*, the first infected cortical cells have been described to collapse, while underneath infected cells remain intact and divide to give rise to the nodule primordium (Bonaldi et al., 2011; Arrighi et al., 2012). Similarly, in *S. rostrata*, intercellular colonization at lateral root base is associated with local cortical cell death and ROS production (D’Haeze et al., 2003). These data provide directions to further investigate the potential link between *AeCRK*, ROS, and early infection. However, at present, it is not known whether the phenotype observed in *crk* mutants reflects the first stage where *AeCRK* intervenes in the symbiotic pathway. Indeed, it cannot be excluded that the phenotype is due to mutations in the *crk-1* and *crk-2* mutants that do not completely inhibit CRK function or is caused by a functional redundancy with another member of the *CRK* cluster to which *AeCRK* belongs (Quilbé et al., 2021). The latter has for instance been observed for the symbiotic receptor gene *LYK3* within the *LYK* cluster in *M. truncatula* (Limpens et al., 2003). In addition, the expression of *AeCRK* throughout nodulation suggests that this gene is also involved in later stages. A similar involvement throughout nodulation has been shown for the symbiotic receptor genes, *NFP*, *LYK3*, and *DMI2*, in *M. truncatula* (Limpens et al., 2003, 2005; Arrighi et al., 2006). We showed that *AeCRK* induction following inoculation required the conserved Nod signaling pathway. Since, downstream of this signaling pathway, *NIN* is known to regulate many rhizobial-induced genes, it is possible that *AeCRK* is part of the *NIN* regulon (Liu et al., 2019). However, rhizobial induction of nodulin genes, including *AeNIN*, was completely abolished in the *crk* mutant, indicating that *AeCRK* is required for the symbiotic signaling process. A reconciling hypothesis is that *AeCRK* expression depends on *AeNIN* and that AeCRK activity both increases signaling to enhance symbiotic responses and promote rhizobial infection (Figure 10). Such dual commitment has already been proposed for the symbiotic receptors LjEPR3 and LjRINRK in *L. japonicus*, highlighting the tight link between symbiotic signaling and rhizobial infection (Li et al., 2019; Kawaharada et al., 2015). Continuing research on this receptor-kinase will likely reveal new and exciting features of rhizobial symbiosis in *A. evenia* and legumes in general.

## Materials and methods

### Plant material and mutant analysis

All *A. evenia* nodulation mutants characterized in this study were obtained from the phenotypic screen of an EMS-mutagenized population derivated from the reference CIAT22838 line (Quilbé et al., 2021). Mutant characteristics are detailed in Supplemental Table S1. For the A21 and E26 mutants, genetic analyses (genetic determinism and allelism tests) and sequencing approaches (genotyping and mapping-by-sequencing) were performed as indicated in Quilbé et al. (2021).

For seed germination, seeds were scarified for 40 min with sulfuric acid (96% v/v) and rinsed with distilled water; germination was induced over night with 0.01% (v/v) ethrel (BAYER) as described earlier (Chaintreuil et al., 2016).

### Plant nodulation and nitrogenese enzyme activity

Standard nodulation tests were performed using *Bradyrhizobium* ORS278 and ORS285, and analysis of the infection process was done using the derivative strains ORS278-GUS or ORS285-GUS (Bonaldi et al., 2011; Giraud et al., 2007). Bacterial culture, root inoculation, and nitrogenase enzyme activity (through the measurement of acetylene reducing activity) as already published (Arrighi et al., 2012). Standard nodulation tests were performed in covered tubes to protect roots from light, except for nodulation kinetics, in order to facilitate nodule number counting. For phenotypic analysis, multiple plant mutants were analyzed in one experiment and compared to one series of WT control plants. Consequently, in some figures of the supplementary information (Supplemental Figures S3–S8 and S11–S16), presented data and photos for the WT control plants are from the same series of WT plants.

### Plant mycorrhization

Germinated seeds were placed on Petri dishes with solid Murashige and Skoog medium (Murashige and Skoog, 1962) to allow root development for 4 days in an in vitro growth chamber. Plants were then transferred by bulks of five individuals in 12 × 12 square pots containing a 1:9 mixture of compost (Nehaus N2) and sand, inoculated with *R. irregularis* DAOM197198 (Agronutrition, Carbonne, France) sterile spores (100 spores per plant), and cultured for 8 weeks in an in vitro growth chamber (28°C, 16-h light regime with light intensity = 130 µE, and 70% humidity). Plants were watered twice a week with a modified Hoagland solution containing 10-µM phosphate as previously described (Gherbi et al., 2008).

To assess and quantify fungal root colonization, roots from pools of five plants per line were collected and cut in 1-cm root fragments. Randomized root fragments were stained using the ink–vinegar method (Chabaud et al., 2002). Quantification of the number of fungal infection points was realized on 15 root fragments/pool using the Myco-Calc method (Trouvelot et al., 1986; htpps://www2.dijon.inra.fr/mychintec/Mycocalc-prg/download.html). Two mycorrhization parameters were evaluated: the frequency (F% = number of mycorrhized fragments/total number)*100) and the intensity (m% =  (95n_5_ + 70n_4_ + 30n_3_ + 5n_2_ + n_1_)/number of mycorrhized fragments, where n_5_ = number of fragments rated 5; n_4_ = number of fragments 4, etc.).

### Light and confocal microscopy

For analysis of the root system architecture, roots were scanned (with an EPSON GT-15000 scanner) and measures of root length or density performed with the Optimas version 6.1 software (Media Cybernitics, Silverspring, MD, USA). For the nodulation kinetics, roots were observed with a binocular loupe at 0, 7, 14, and 21 dpi and the number of bumps, white and pink nodules were recorded. Nontreated, nodulated, and mycorrhized roots were observed using a stereo-macroscope (Niko AZ100, Champigny-sur-Marne, France) and pictures taken with the Nikon Advanced software. For root hair lengths, measurements were taken with ImageJ version 1.53e software (http://imageJ.nih.gov/ij). Bacterial infection of plant tissue was analyzed using 70-µm-thick vibratome (Leica VT1000S) sections of fresh material. In case of infection with strain ORS278-GUS, sections were stained with X-gluc (Fabre et al., 2015) and observed using a Nikon macroscope. To analyze the infection process, 70-µm root sections of plants inoculated with ORS278 or ORS285 were stained with Syto13 and Calcofluor White and visualized by confocal microscopy with the following excitation laser/emission cutoffs: 405/410–500 nm (Calcofluor White: intensity 5%–11%; gain: 500–800), 488/493–525 nm (Syto-13: intensity 5%–11%; gain: 500–750), 555/560–630 nm (autofluorescence: 6%–11%; gain: 500–800). Analysis and photos were taken using a confocal laser-scanning microscope (Carl Zeiss LSM 700; Jen, Germany), and obtained images were processed using the Carl Zeiss Confocal Microscope software.

### In silico gene expression and co-expression analysis

Expression patterns for genes of interest were obtained using the *A. evenia* gene atlas available at the AeschynomeneBase (http://aeschynomenebase.fr/content/gene-expression). To identify genes co-expressed with *AeNSP2*, a hierarchical clustering analysis using Pearson correlation was performed. Raw RNAseq data available for *A. evenia* were converted into fragments per kilobase of exon per million mapped fragments. After filtering data to remove genes with low expression levels, a hclust hierarchical clustering was performed on a distance matrix calculated with the Pearson method included in the AMAP package (version 0.8.17). The clustering tree was initially split into 12 and then 100 clusters. The one containing *AeNSP2* was analyzed and co-expressed genes subsequently validated by RT–qPCR are listed in Supplemental Table S4. Homology and orthology were inferred for *A. evenia* genes with model legumes using previously ORTHOFINDER-delineated groups of orthologous genes (Quilbé et al., 2021), and with *A. thaliana* genes using the BLAST tool on the TAIR web portal (https://www.arabidopsis.org).

### RNA extraction and expression studies

All shown experiments contained at least three biological replicates. To study the gene regulation by nitrogen and *AeNSP2* at 10-day postgermination, we took for each mutant line and nitrogen concentration (0, 0.5, and 5 mM KNO_3_) a pool of three plant roots for analysis and the experiment was repeated 3 times. For the nodulation kinetics, a pool of three plant roots per line and time point (0, 2, and 4 days after inoculation with *Bradyrhizobium* ORS278) was done and the experiment was repeated 3 times. For the mycorrhization tests, at 8-week postinoculation, for each mutant line and condition (inoculated or not with *R. irregularis*), a pool of five plant roots was collected and analyzed. The experiments were repeated 3, 4, or 7 times, as detailed in figure legends, to ensure data robustness.

Total RNA extraction from roots, RT–qPCR were performed as described (Fabre et al., 2015; Gully et al., 2018). Expression levels were normalized with the *AeEF1-α* and *AeUbi* reference genes. Gene-specific primers were designed with Beacon Designer (Premier Biosoft) using the annotated *A. evenia* genome for plant genes, while primer sequences were obtained from the literature data for fungi genes (Supplemental Table S5).

For the study of splicing variants in the *crk-2* mutant, cDNAs were amplified using the primers CRKexon3-F (5′-GGGTGGGACAAGTGATGAAA-3′) and CRKexon6-R (5′-CCAGCATCAGTACCCCAAAG-3′), purified using the QIAquick gel extraction kit (Qiagen, Hilden, Germany) and then sequenced.

### Kinase activity

The predicted intracellular region of CRK (S288–Y668) was amplified using primers AeCRKkin-BglF (5′-CCCAGATCTTCAAAAATAAGAAGAAGATTAAAAGG-3′) and AeCRKkin-NotR (5′-ATTTGCGGCCGCTTAATAGAAAGTAGAAACAGAGC-3′) and cloned in the pCDFDuet-1 vector (Novagen, Vadodara, India), which had been modified to include a glutathione-S-transferase (GST) sequence after the encoded 6 × His residues. The G354E mutation (corresponding to the *crk-2* allele) was created by site-directed mutagenesis using primers AeCRKmutJ42F (5′-GAAACCAAGCTTGAAGAAGGTGGCTTC3′) and AeCRKmutJ42R (5′-GAAGCCACCTTCTTCAAGCTTGGTTTC3′). The resulting plasmids, pCDFDuet-6hisGST-CRKkin and pCDFDuet-6hisGST-CRKkin-G354E, were checked by sequencing and then transformed into *E. coli* Rosetta/DE3 (Novagen). The fusion proteins were expressed and purified on Glutathione-Sepharose4B (Amersham Biosciences Amersham, UK) as described (Klaus-Heisen et al. 2011). Kinase activity assays used the same conditions, except that no radioactivity was used and the concentration of ATP was increased to 20 µM. Autophosphorylation was detected by western blotting using anti-phospho-Thr antibodies as described (Klaus-Heisen et al. 2011), except that a dilution of 1/5,000 was used. Duplicate gels were stained with Coomassie blue protein stain. The protein purifications and kinase assays were repeated at least twice. Two dead kinase proteins from *M. truncatula*, GST-NFPkin (Arrighi et al., 2006) and GST-LYK3kin-G334E (Klaus-Heisen et al., 2011) and GST were used as controls. Quantification of the bands used the volume tools of Image Lab version 6.0 (BioRad Laboratories, Hercules, CA, USA).

### Statistical analysis

All measurements and gene expression levels were compared between the WT and mutant lines or between the control condition and tested conditions using a Kruskal–Wallis test or Student’s *t* test with R package.

### Accession numbers

The mapping-by-sequencing data generated for the *nin-1* mutant in this study were deposited in the NCBI database under BioProject ID: PRJNA727694. The *A. evenia* gene identifiers are shown in Supplemental Tables S1, S4, and S5.

## Supplemental data

The following materials are available in the online version of this article.

**Supplemental Figure S1.** Identification of a *nin* mutant allele by mapping-by-sequencing.

**Supplemental Figure S2.** Comparison of root hair development and root system architecture in the WT line, the *ccamk-3* mutant, and the four allelic *nsp2* mutants of *A. evenia*.

**Supplemental Figure S3.** Nodulation properties of the *pollux* mutants with *Bradyrhizobium* strain ORS278.

**Supplemental Figure S4.** Nodulation properties of the *ccamk* mutants with *Bradyrhizobium* strain ORS278.

**Supplemental Figure S5.** Nodulation properties of the *cyclops* mutants with *Bradyrhizobium* strain ORS278.

**Supplemental Figure S6.** Nodulation properties of the *nsp2* mutants with *Bradyrhizobium* strain ORS278.

**Supplemental Figure S7.** Nodulation properties of the *nin* mutants with *Bradyrhizobium* strain ORS278.

**Supplemental Figure S8.** Nodulation properties of the *crk* mutants with *Bradyrhizobium* strain ORS278.

**Supplemental Figure S9.** Abnormal splicing of *AeCRK* transcripts in the *crk-1* mutant.

**Supplemental Figure S10.** Analysis of the kinase activity of AeCRK and the G354 mutant protein, corresponding to the mutation in *crk-2*.

**Supplemental Figure S11.** Nodulation properties of *pollux* mutants with *Bradyrhizobium* strain ORS285.

**Supplemental Figure S12.** Nodulation properties of a *ccamk* mutant with *Bradyrhizobium* strain ORS285.

**Supplemental Figure S13.** Nodulation properties of *cyclops* mutant with *Bradyrhizobium* strain ORS285.

**Supplemental Figure S14.** Nodulation properties of a *nsp2-2* mutant with *Bradyrhizobium* strain ORS285.

**Supplemental Figure S15.** Nodulation properties of *nin* mutants with *Bradyrhizobium* strain ORS285.

**Supplemental Figure S16.** Nodulation properties of *crk* mutants with *Bradyrhizobium* strain ORS285.

**Supplemental Figure S17.** Expression of mycorrhization-related genes in the *A. evenia* WT line.

**Supplemental Table S1.** Molecular and phenotypic properties of *A. evenia* nodulation mutants.

**Supplemental Table S2.** Genetic determinism of *nin* mutants.

**Supplemental Table S3.** Allelism tests performed on *nin* mutants.

**Supplemental Table S4.** Expression data of *A. evenia* genes induced or repressed during nodulation.

**Supplemental Table S5.** List of genes with the primers used for gene expression analysis.

## Supplementary Material

kiac325_Supplementary_Data

## Notes

J.Q., N.N., M.P., R.G., J.C., N.H.A., and F.G. designed and performed the experiments, and analyzed the data. D.G. and C.K. contributed to gene analyses. J.F.A. conceived the research plan and prepared the manuscript draft. N.N., J.C., and E.G. critically revised the manuscript, and all authors approved the final version of the manuscript.

The author responsible for distribution of materials integral to the findings presented in this article in accordance with the policy described in the Instructions for Authors (https://academic.oup.com/plphys/pages/general-instructions) is: Jean-François Arrighi (jean-francois.arrighi@ird.fr).
