# Supplementary material for: A mutant-based analysis of the establishment of Nod-independent symbiosis in the legume Aeschynomene evenia
Source: Plant Physiol. 2022 Jul 25;190(2):1400–17. doi: 10.1093/plphys/kiac325 (PMC9516736; doi:10.1093/plphys/kiac325)
Supplement: kiac325_Supplementary_Data [file kiac325_supplementary_data.zip › Supplemental Figures S1S17 vf.pdf]

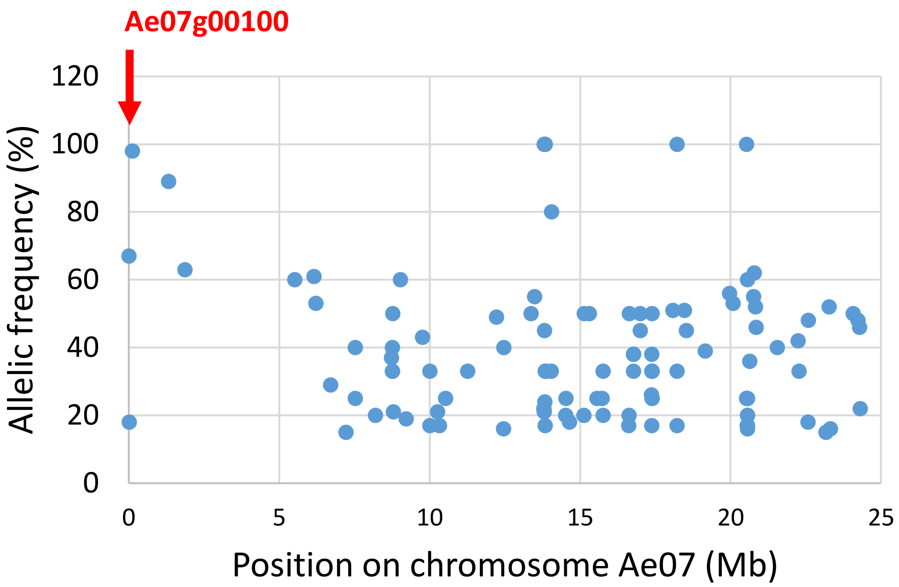

Supplemental Figure S1. Identification of a *nin* mutant allele by Mapping-by-Sequencing. The frequency of the EMS-induced mutations in a bulk of mutant backcrossed F2 plants was obtained for the A21 mutant of *A. evenia* using the Mapping-by-Sequencing approach. A genetic linkage corresponding to a shift of the allelic frequency is visible at the beginning of chromosome Ae07. The SNP identified in the Ae07g00100 (*AeNIN*) gene is indicated by a red arrow and represents the putative causal mutation.

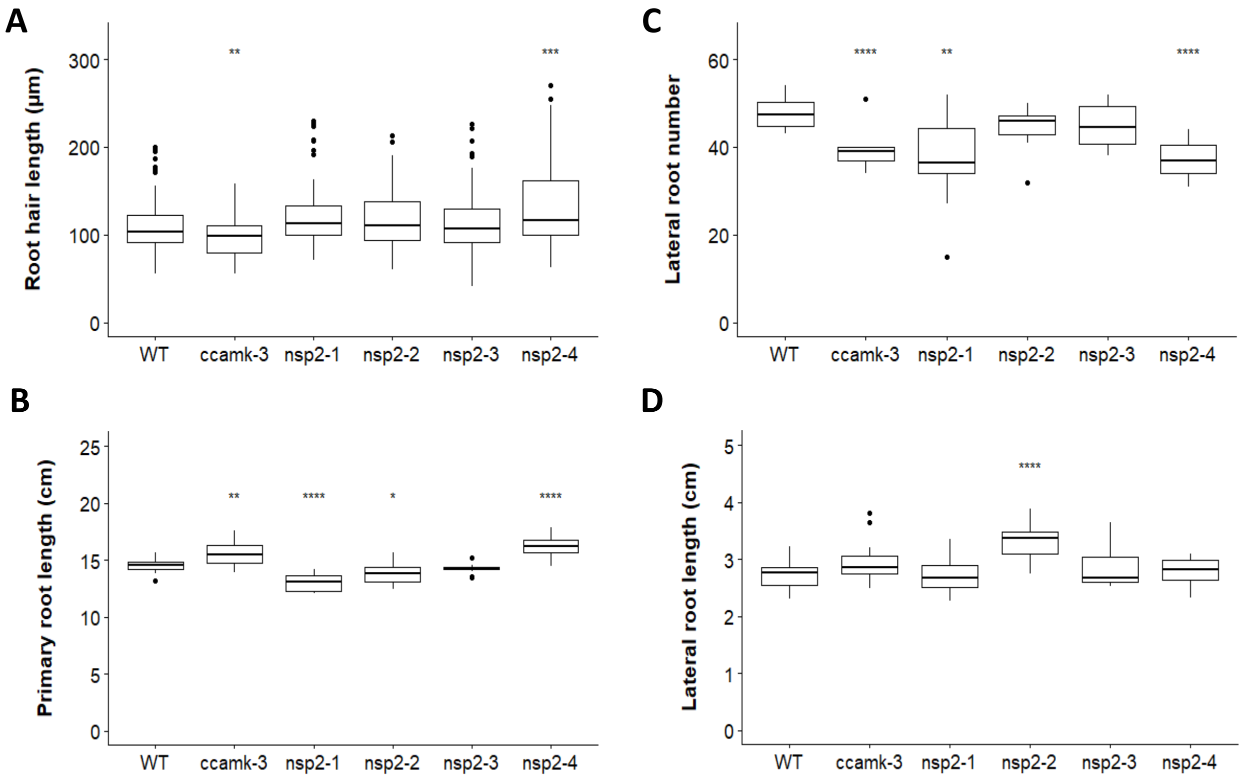

Supplemental Figure S2. Comparison of root hair development and root system architecture in the wild-type (WT) line, the *ccamk-3* mutant and the four allelic *nsp2* mutants of *A. evenia*. (**A**) Root hair length. (**B**) Primary root length. (**C**) Number of lateral roots on a segment of 3 cm in the upper part of the primary root. (**D**) Lateral root length on the same segment of 3 cm of the primary root. Box plots result in (**A**) from the measurement of 100 root hairs from 5 plants/line and in (**B**-**D**) from the measurement of primary and lateral roots from 12 plants/line. The rectangle spans the first quartile to the third quartile; the central segment shows the median; and the whiskers above and below the box show the locations of the maximum and minimum value, respectively. Outliers are represented by dots. *P < 0.05, **P < 0.01, ***P < 0.001, ****P < 0.0001 significant differences between WT and each mutant using the Student's *t* test.

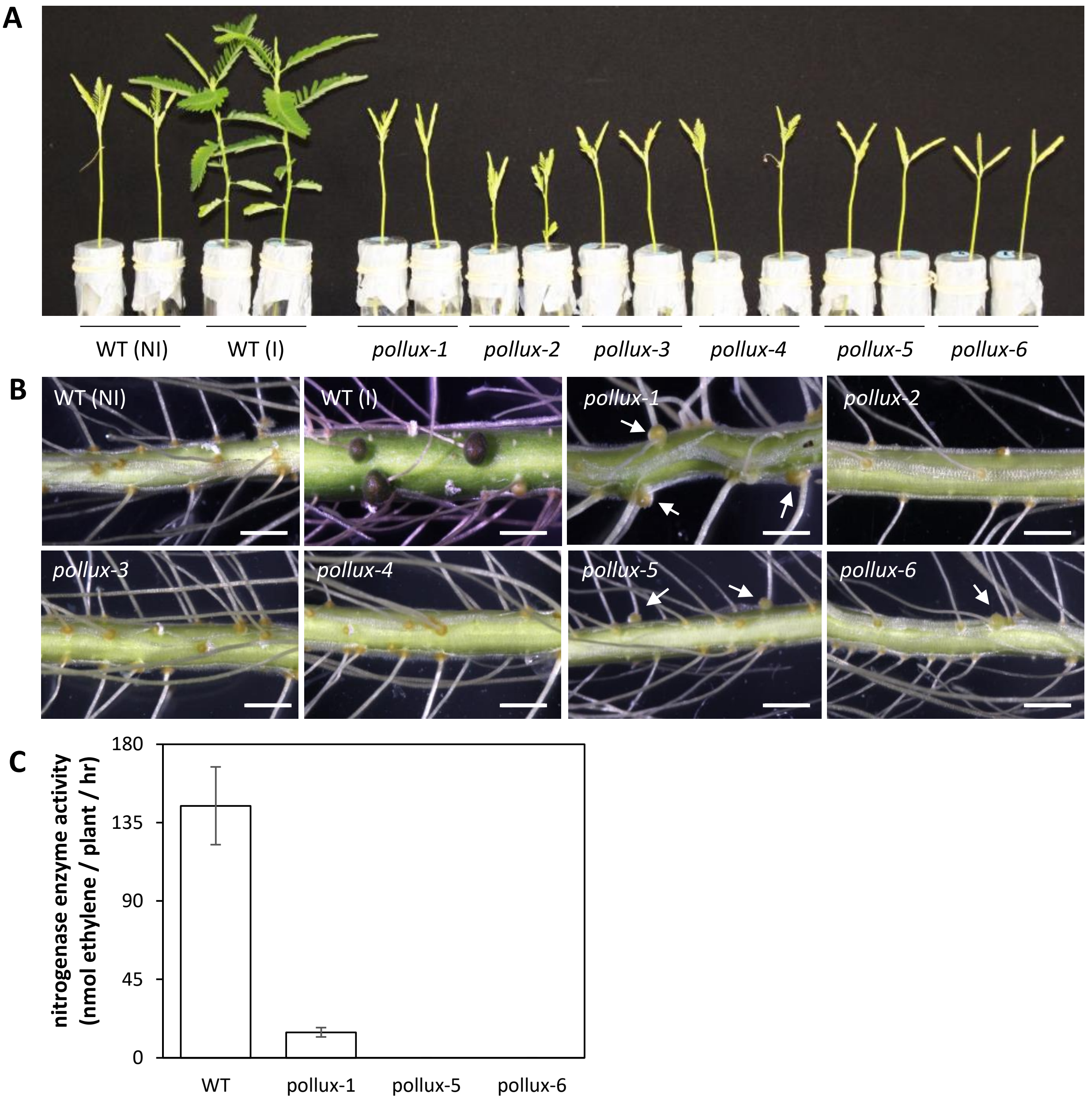

Supplemental Figure S3. Nodulation properties of the *pollux* mutants with *Bradyrhizobium* strain ORS278. (**A**) Comparison of the growth of the plants (aerial part), non-inoculated (NI) or inoculated (I) at 21 dpi. (**B**) Roots of the wild-type (WT) line and of the different mutants presenting or not nodules under different stages of development. Arrows indicate bumps, white or pink nodules. 20 plants/line were analyzed. Scale bars: 2 mm. (**C**) Acetylene-reducing activity (ARA) on plants at 21 dpi. Error bars represent s.d. (n=6). Note that the same WT plants were used for multiple comparisons with allelic mutant series from one experiment.

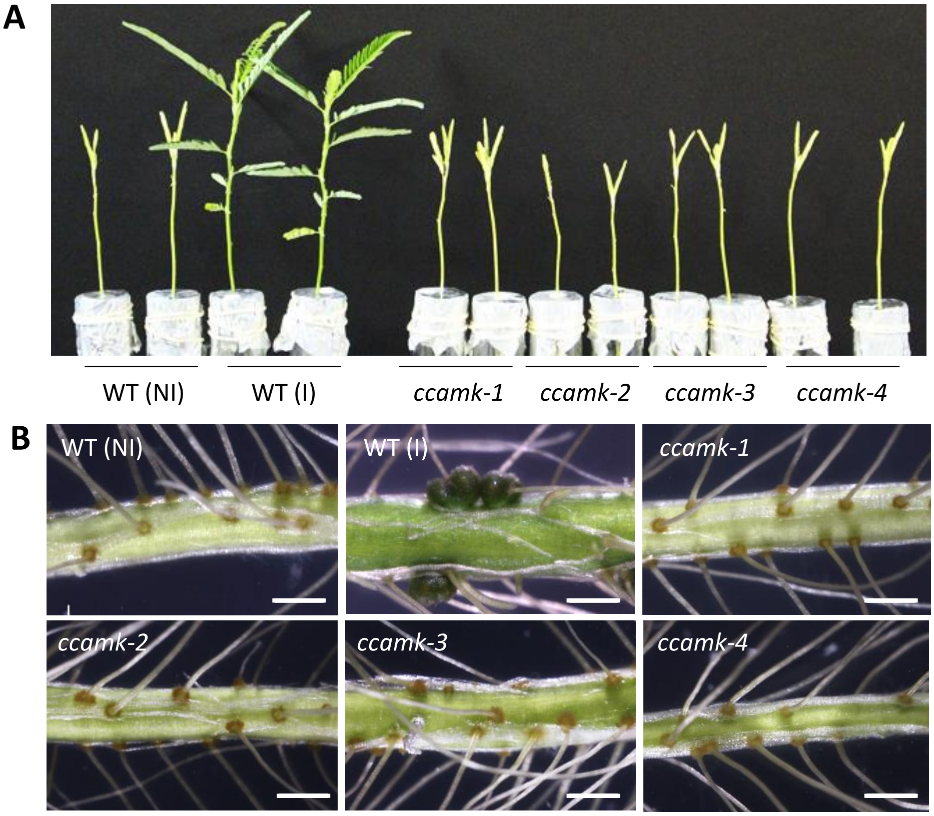

Supplemental Figure S4. Nodulation properties of the *ccamk* mutants inoculated with *Bradyrhizobium* strain ORS278. (**A**) Comparison of the growth of the plants (aerial part), non-inoculated (NI) or inoculated (I) at 21 dpi. (**B**) Roots of the wild-type (WT) line presenting or not nodules, and of the different mutants. 20 plants/line were analyzed. Note that the same control plants were used for comparison with different allelic mutant series from a single experiment (Figures S4, S6 and S7). Scale bars: 2 mm.

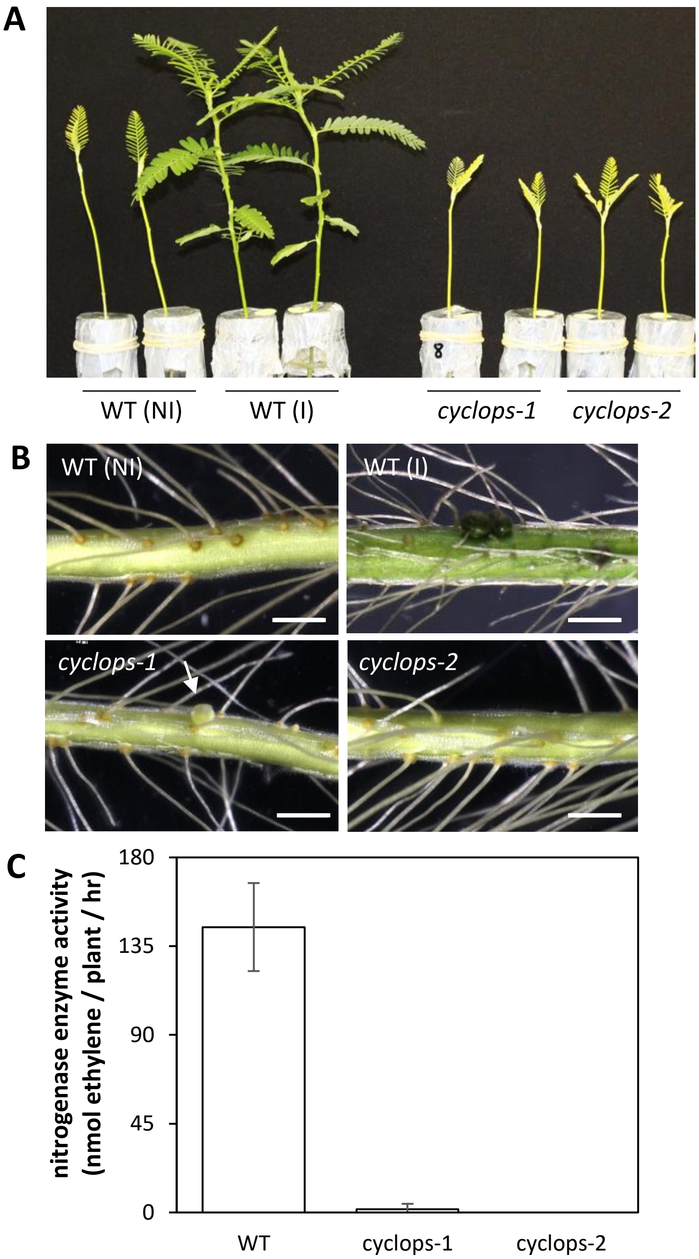

Supplemental Figure S5. Nodulation properties of the *cyclops* mutants with *Bradyrhizobium* strain ORS278. (**A**) Comparison of the growth of the plants (aerial part), non-inoculated (NI) or inoculated (I) at 21 dpi. (**B**) Roots of the wild-type (WT) line and of the two allelic mutants presenting or not nodules. The arrow shows a white nodule. 20 plants/line were analyzed. Scale bars: 2 mm. (**C**) Acetylene-reducing activity (ARA) on plants at 21 dpi. Error bars represent s.d. (n=6).

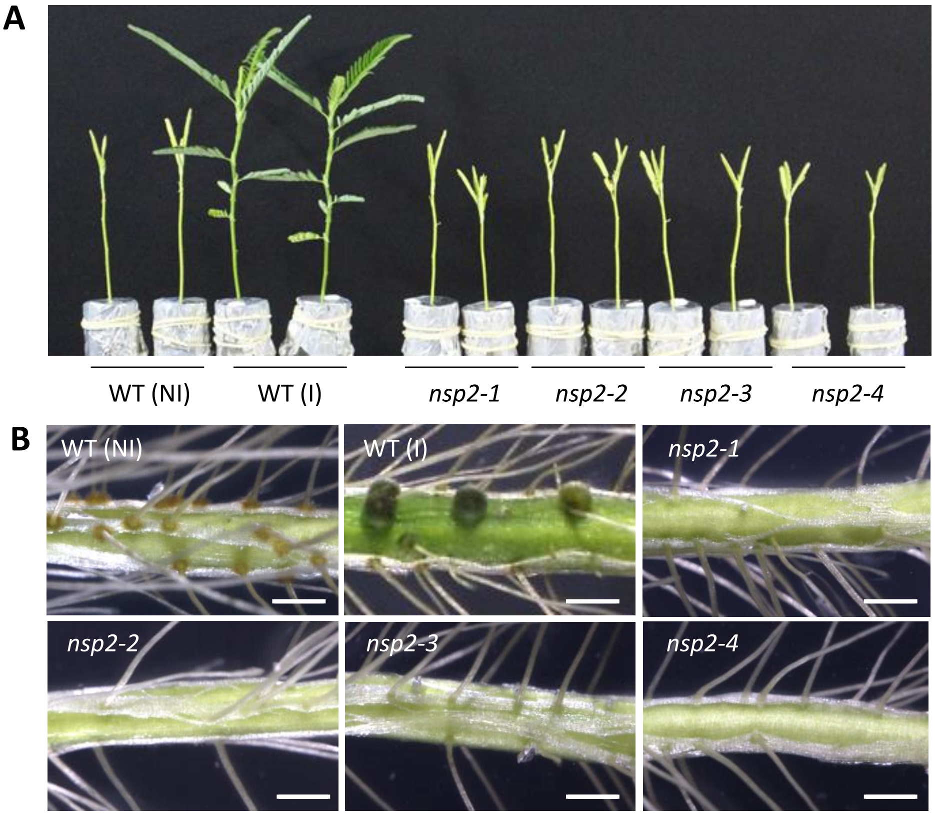

Supplemental Figure S6. Nodulation properties of the *nsp2* mutants with *Bradyrhizobium* strain ORS278. (**A**) Comparison of the growth of the plants (aerial part), non-inoculated (NI) or inoculated (I) at 21 dpi. (**B**) Roots of the wild-type (WT) line presenting or not nodules, and of the different *nsp2* mutants that are characterized by the absence of axillary root hair crown at the base of lateral roots. 20 plants/line were analyzed. Note that the same control plants were used for comparison with different allelic mutant series from a single experiment (Figures S4, S6 and S7). Scale bars: 2 mm.

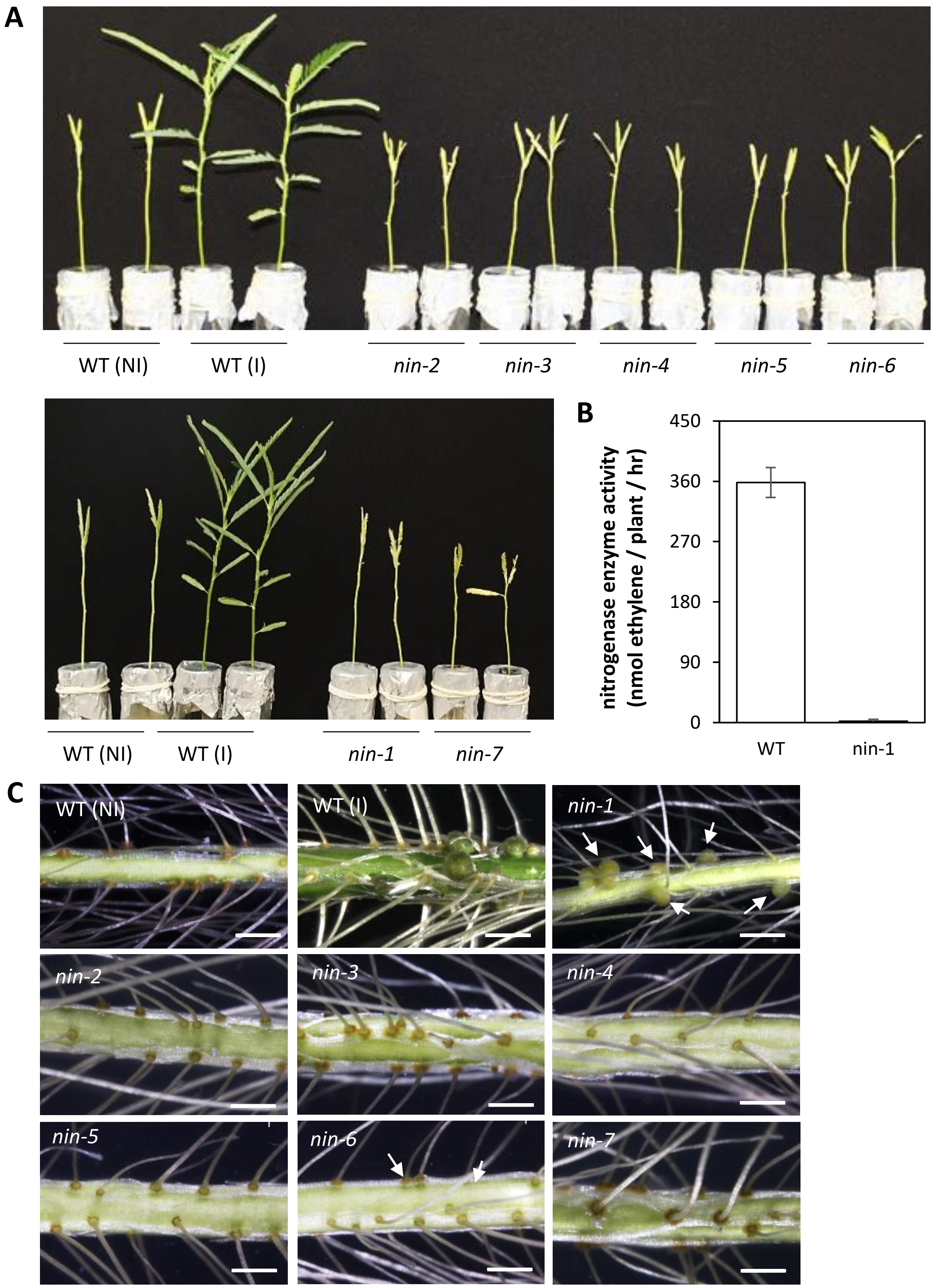

Supplemental Figure S7. Nodulation properties of the *nin* mutants with *Bradyrhizobium* strain ORS278. (**A**) Comparison of the growth of the plants (aerial part), non-inoculated (NI) or inoculated (I) at 21 dpi. (**B**) Acetylene-reducing activity (ARA) on plants at 21 dpi. Error bars represent s.d. (n=6). (**C**) Roots of the wild-type (WT) line and of the different mutants presenting or not nodules. Arrows indicate white nodules. 20 plants/line were analyzed. Note that the same control plants could be used for comparison with different allelic mutant series from a single experiment (Figures S4, S6 and S7). Scale bars: 2 mm.

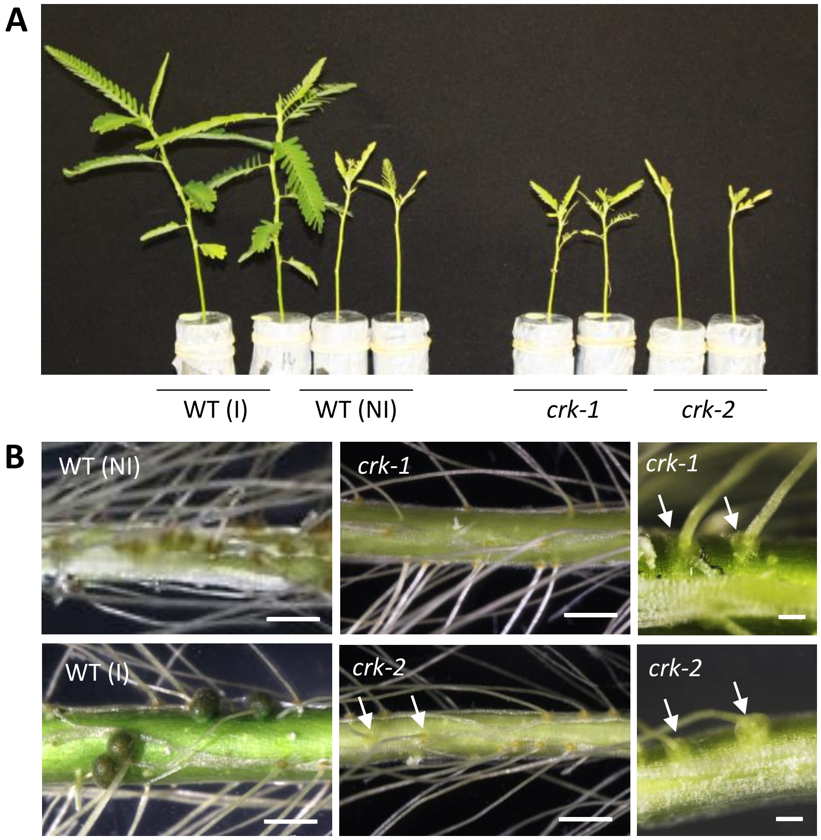

Supplemental Figure S8. Nodulation properties of the *crk* mutants with *Bradyrhizobium* strain ORS278. (**A**) Comparison of the growth of the plants (aerial part), non-inoculated (NI) or inoculated (I) at 21 dpi. (**B**) Roots of the wild-type (WT) line and of the two allelic mutants presenting or not bumps. The arrows show small bumps that are best observable by macroscopy (right panel). 20 plants/line were analyzed. Scale bars: 2 mm, except right panel: 700 µm.

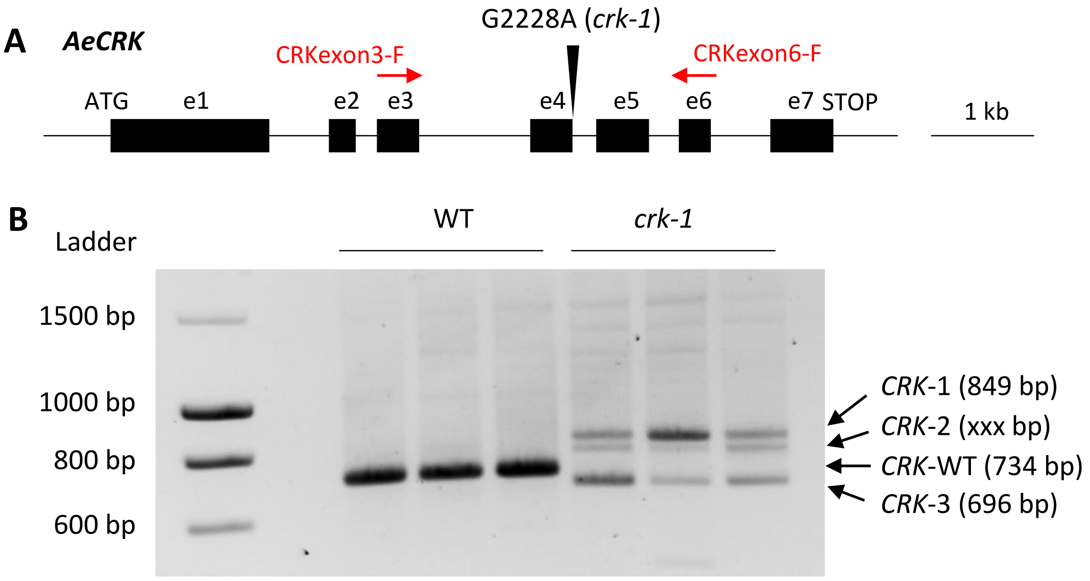

C

CRK-WT (normal splicing)
TGAAAAGCTTCTTGTCTATGAGTACATGGCCAACACTAGTCTTGATGCTTTCCTCTTTGATCCAACCAAGCGTAGAAAGCTTGAT

CRK-1 (unspliced form)
TGAAAAGCTTCTTGTCTATGAGTACATGGCCAACACTAGTCTTGATGCTTTCCTCTTTGATTAGCTCTTCCTAACCCTCTATCTATGT
TATAGACATTTGATATTTTGATTCAAGGAGAGCTTCCTAATCTAAATTCTGAAGCCATCTTTTCTACTTTTACCCATGTAAAGCAGATC
CAACCAAGCGTAGAAAGCTTGAT

CRK-2 (partially spliced form)
TGAAAAGCTTCTTGTCTATGAXXXXXXXXXXXXXXXXXXXXXXXXXXXXXXXXXXXXXXXXXXXXXXXXXXXXXXXXXXXXXXXXXXXXX
XXXXXXXXXXXXXXXXXXXXXXXXXXXXXXXXXXXXXXXXXXXXXXXXXXXXXXXXXCCCATGTAAAGCAGATCCAACCAAGCGTAGA
AAGCTTGAT

CRK-3 (truncated form)
TGAAAAGCTTCTTGTCTATGAATCCAACCAAGCGTAGAAAGCTTGAT

Supplemental Figure S9. Abnormal splicing of *AeCRK* transcripts in the *crk-1* mutant. (**A**) The *AeCRK* gene contains seven exons (e) represented by black boxes and six introns represented by black lines. The G2228A mutation present in the *crk-1* mutant is at the junction of exon 4 and intron 4. Red arrows correspond to the primers used for PCR amplication of *AeCR*K transcripts. (**B**) PCR amplification profiles obtained using the primers CRKexon3-F and CRKexon6-R on cDNAs from roots of uninoculated wild-type (WT) and *crk-1* mutant (three idependent samples loaded for each). Note the presence of a single band at the expected size for the WT (734-bp) and of three bands for the *crk-1* mutant. (**C**) Comparison of *AeCRK* transcript sequences between exon 4 (blue) and exon 5 (green) obtained in the WT and *crk-1* mutant. In black, the intron sequence and in red, the G->A mutated splicing site. Note that the major *CRK*-1 band and the minor *CRK*-2 band were sequenced together and full sequence was obtained for the major band.

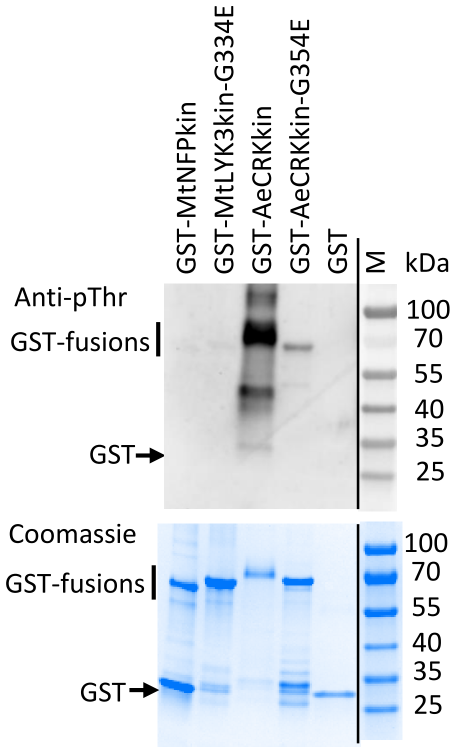

Supplemental Figure S10. Analysis of the kinase activity of AeCRK and the G354 mutant protein, corresponding to the mutation in *crk-2*. GST-fusion proteins of the kinase domains were purified from *E. coli*, assayed with ATP and analyzed by SDS-PAGE and Western blotting using anti-phospho-Thr antibodies. GST fusions of the MtNFPkin and MtLYK3kin-G334E dead kinase proteins were used as controls. The position of purified proteins is indicated by coomassie blue staining. Note that the two proven dead-kinase proteins of *Medicago truncatula*, GST-NFPkin and GST-LYK3kin-G334E, and GST alone showed no reaction to the antibody while the wild-type AeCRK kinase protein was strongly phosphorylated compared to the G354E mutant protein(about 80-fold more).

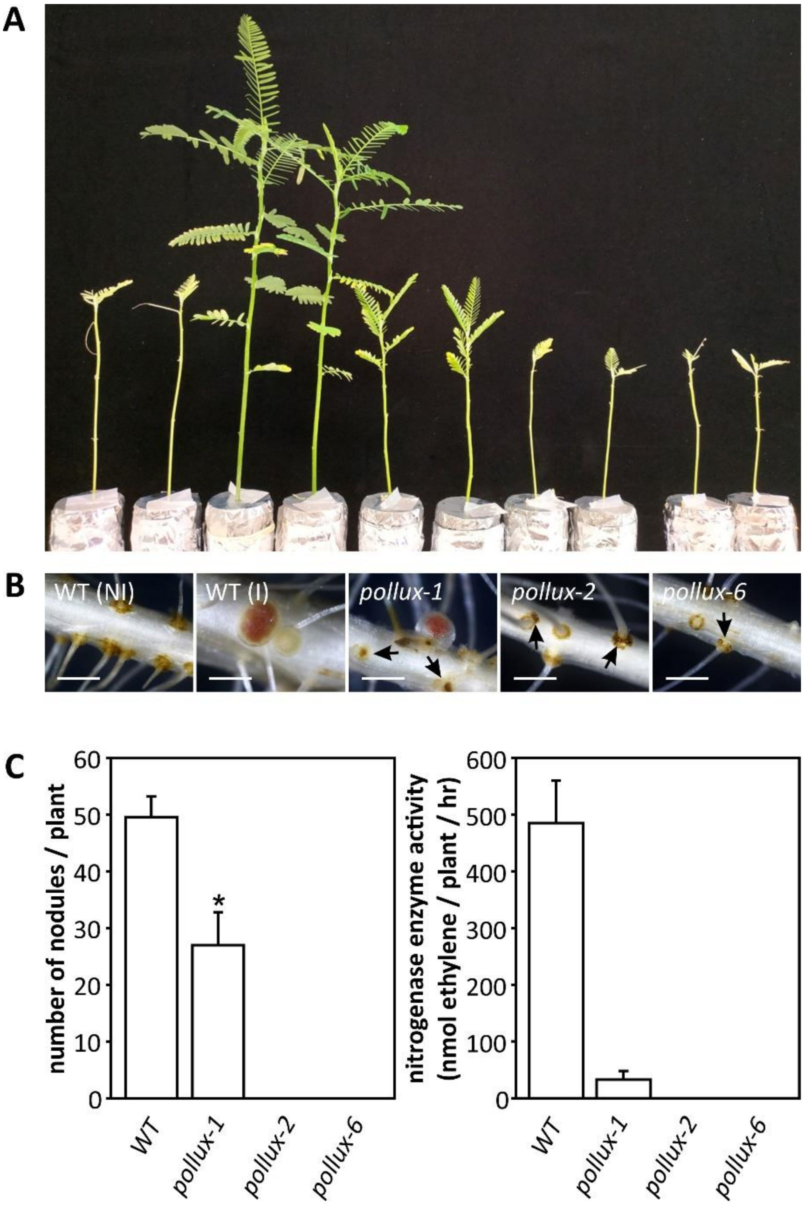

Supplemental Figure S11. Nodulation properties of *pollux* mutants with *Bradyrhizobium* strain ORS285. (**A**) Comparison of the growth of the plants (aerial part), non-inoculated (NI) or inoculated (I) at 21 dpi. (**B**) Roots of the wild-type (WT) and different *pollux* mutants, presenting nodules or not. Arrows indicate brown spots at the base of lateral roots. Minimum of 6 plants/line were analyzed. Scale bars: 2 mm. (**C**) Number of nodules (left) and nitrogenase enzyme activity (right) of plants at 21 dpi. * bar indicate the amount of nodules and bumps on the roots of the *pollux-1* mutant. Error bars represent s.d. (n=6). Note that the same control plants were used for comparison with different mutants from a single experiment (Figures S11 and S15).

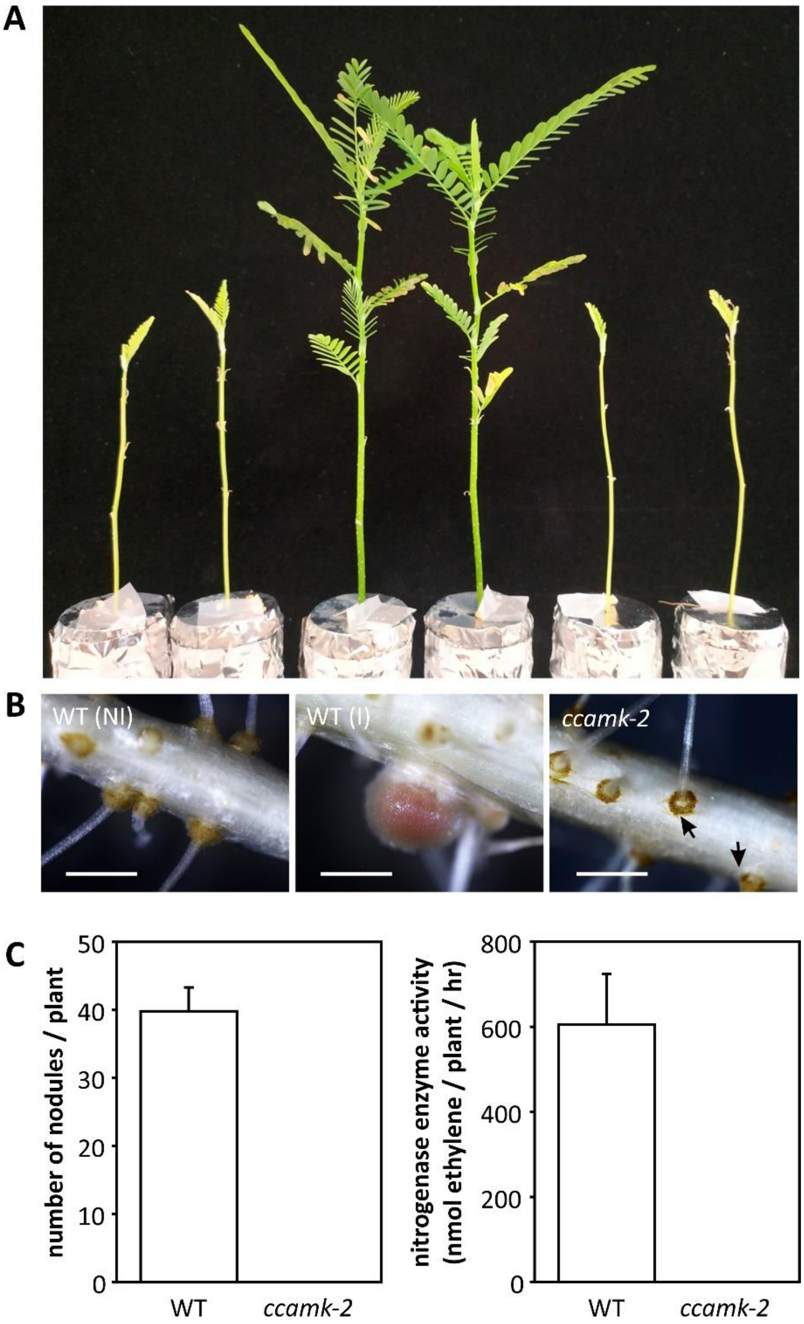

Supplemental Figure S12. Nodulation properties of the *ccamk-2* mutant with *Bradyrhizobium* strain ORS285. (**A**) Comparison of the growth of the plants (aerial part), non-inoculated (NI) or inoculated (I) at 21 dpi. (B) Roots of the wild-type (WT) line and of the *ccamk-2* mutant, presenting or not nodules. Arrows indicate intense browning at the base of lateral roots. Minimal 6 plants/line were analyzed. Scale bars: 2 mm. (**C**) Number of nodules (left) and nitrogenase enzyme activity) (right) of plants at 21 dpi. Error bars represent s.d. (n=6). Note that the same control plants were used for comparison with different mutants from a single experiment (Figures S12-S14 and S16).

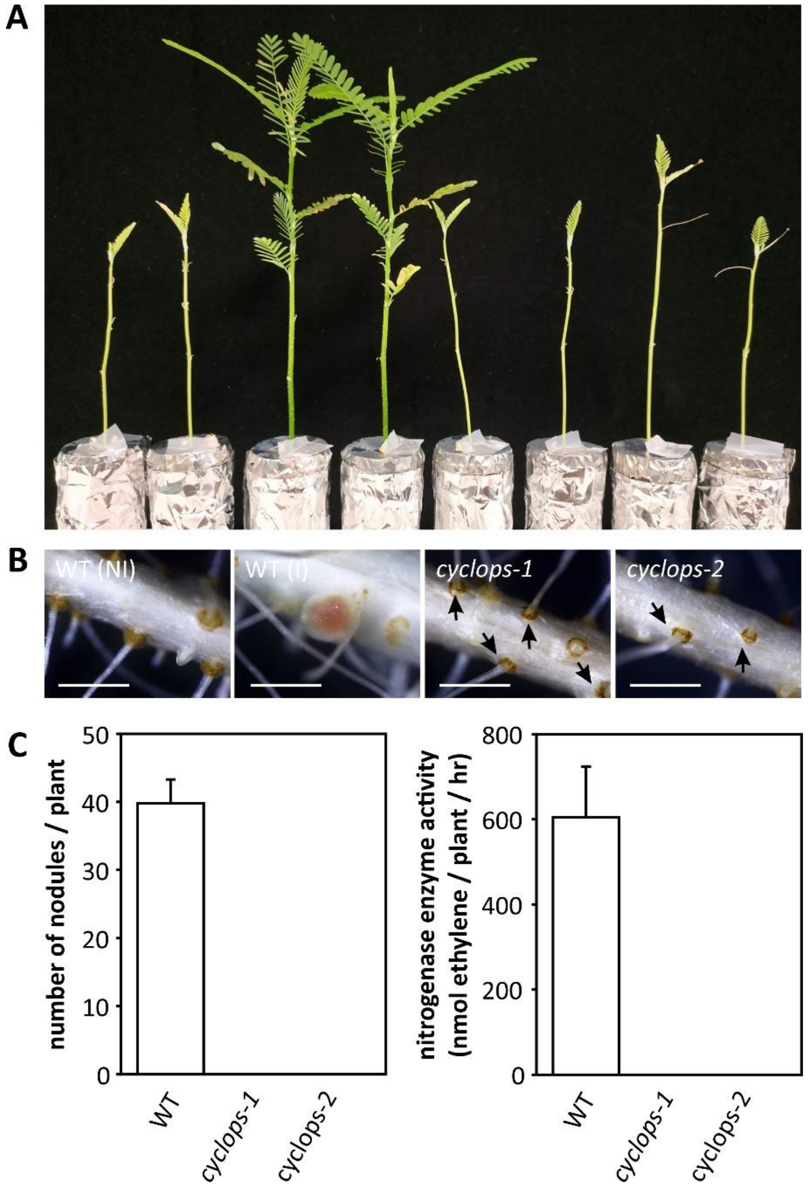

Supplemental Figure S13. Nodulation properties of *cyclops* mutants with *Bradyrhizobium* strain ORS285. (**A**) Comparison of the growth of the plants (aerial part), non-inoculated (NI) or inoculated (I) at 21 dpi. (**B**) Roots of the wild-type (WT) line and *cyclops* mutants, presenting or not nodules. Arrows indicate intense browning at the base of lateral roots. Minimum of 6 plants / line were analyzed. Scale bars: 2 mm. (**C**) Number of nodules (left) and nitrogenase enzyme activity (right) on plants at 21 dpi. Error bars represent s.d. (n=6). Note that the same control plants were used for comparison with different mutants from a single experiment (Figures S12-S14 and S16).

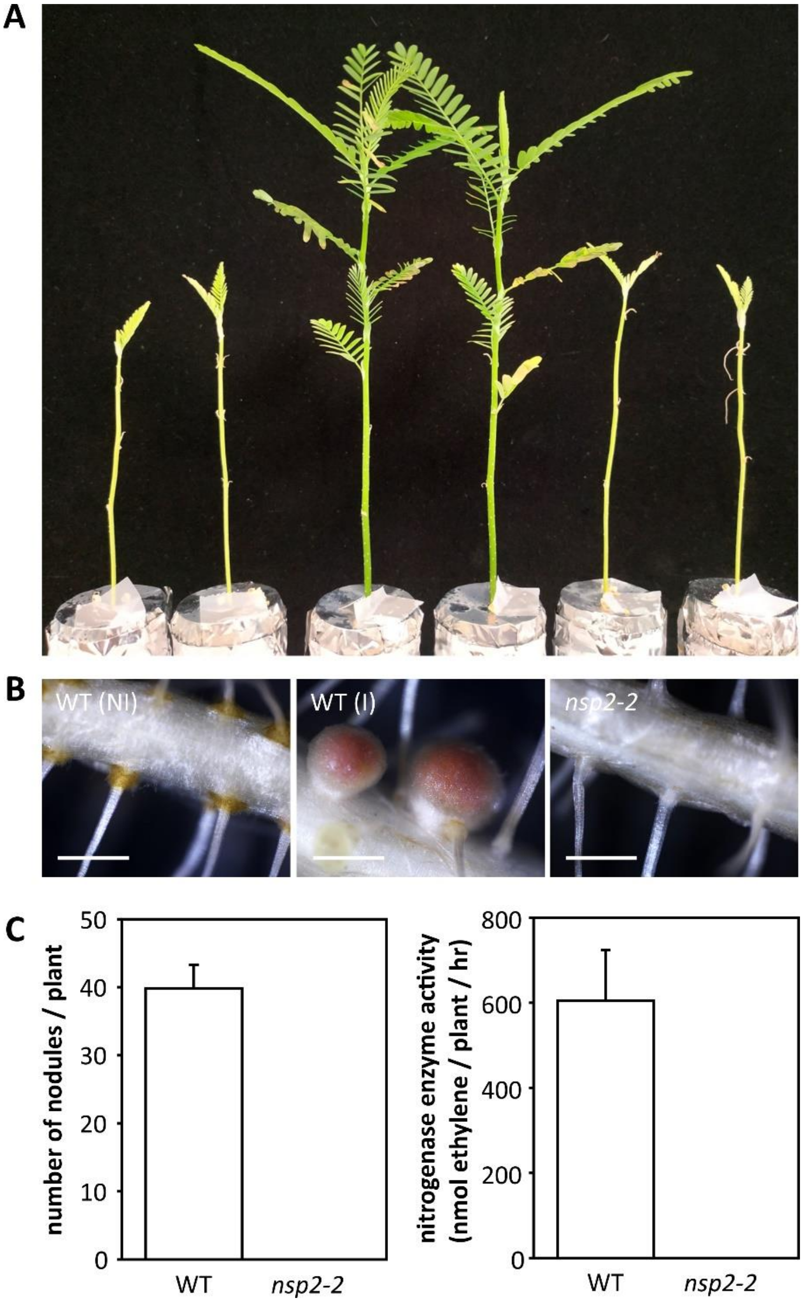

Supplemental Figure S14. Nodulation properties of the *nsp2-2* mutant with *Bradyrhizobium* strain ORS285. (**A**) Comparison of the growth of the plants (aerial part), non-inoculated (NI) or inoculated (I) at 21 dpi. (**B**) Roots of the wild-type (WT) line and of the *nsp2-2* mutant, presenting or not nodules. Minimum of 6 plants/line were analyzed. Scale bars: 2 mm. (**C**) Number of nodules (left) and nitrogenase enzyme activity (right) on plants at 21 dpi. Error bars represent s.d. (n=6). Note that the same control plants were used for comparison with different mutants from a single experiment (Figures S12-S14 and S16).

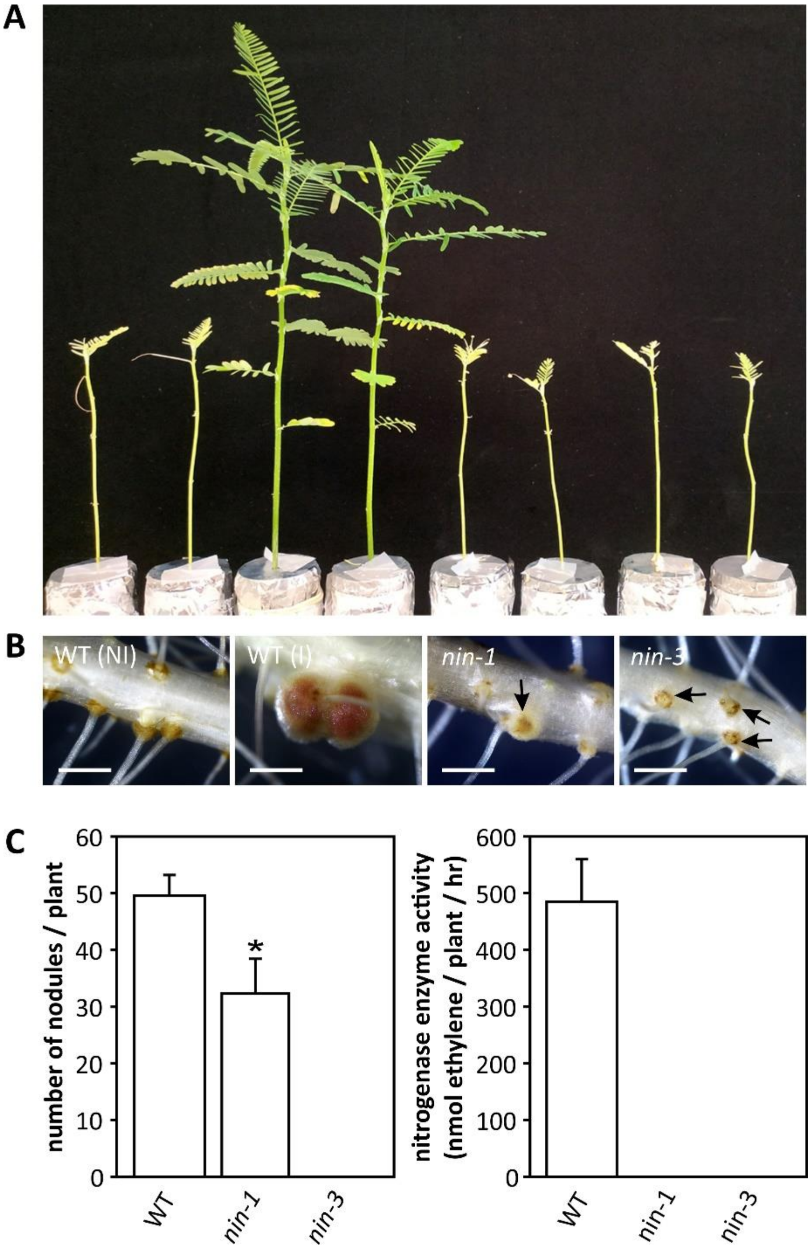

Supplemental Figure S15. Nodulation properties of *nin* mutants with *Bradyrhizobium* strain ORS285. (**A**) Comparison of the growth of the plants (aerial part), non-inoculated (NI) or inoculated (I) at 21 dpi. (**B**) Roots of the wild-type (WT) line and *nin* mutants, presenting or not nodules. Arrows indicate intense browning in bumps at base of lateral roots. Minimum of 6 plants/line were analyzed. Scale bars: 2 mm. (**C**) Number of nodules (left) and nitrogenase enzyme activity (right) on plants at 21 dpi. Error bars represent s.d. (n=6). * indicates the number of bumps on roots of the nin-1 mutant. Note that the same control plants were used for comparison with different mutants from a single experiment (Figures S11 and S15).

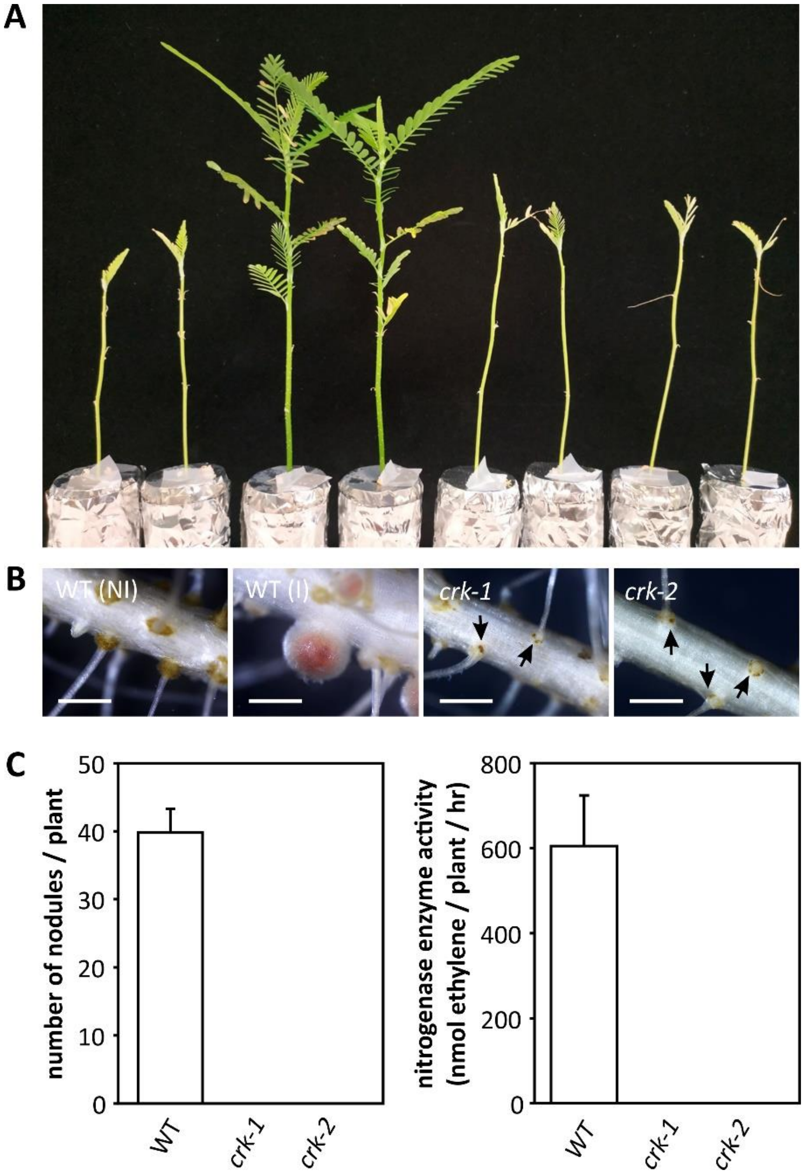

Supplemental Figure S16. Nodulation properties of *crk* mutants with *Bradyrhizobium* strain ORS285. (**A**) Comparison of the growth of the plants (aerial part), non-inoculated (NI) or inoculated (I) at 21 dpi. (**B**) Roots of the wild-type (WT) line and of the mutants, presenting or not nodules. Arrows indicate intense brown spots at the base of lateral roots. Minimum of 6 plants / line were analyzed. Scale bars: 2 mm. (**C**) Number of nodules (left) and nitrogenase enzyme activity (right) on plants at 21 dpi. Error bars represent s.d. (n=6). Note that the same control plants were used for comparison with different mutants from a single experiment (Figures S12-S14 and S16).

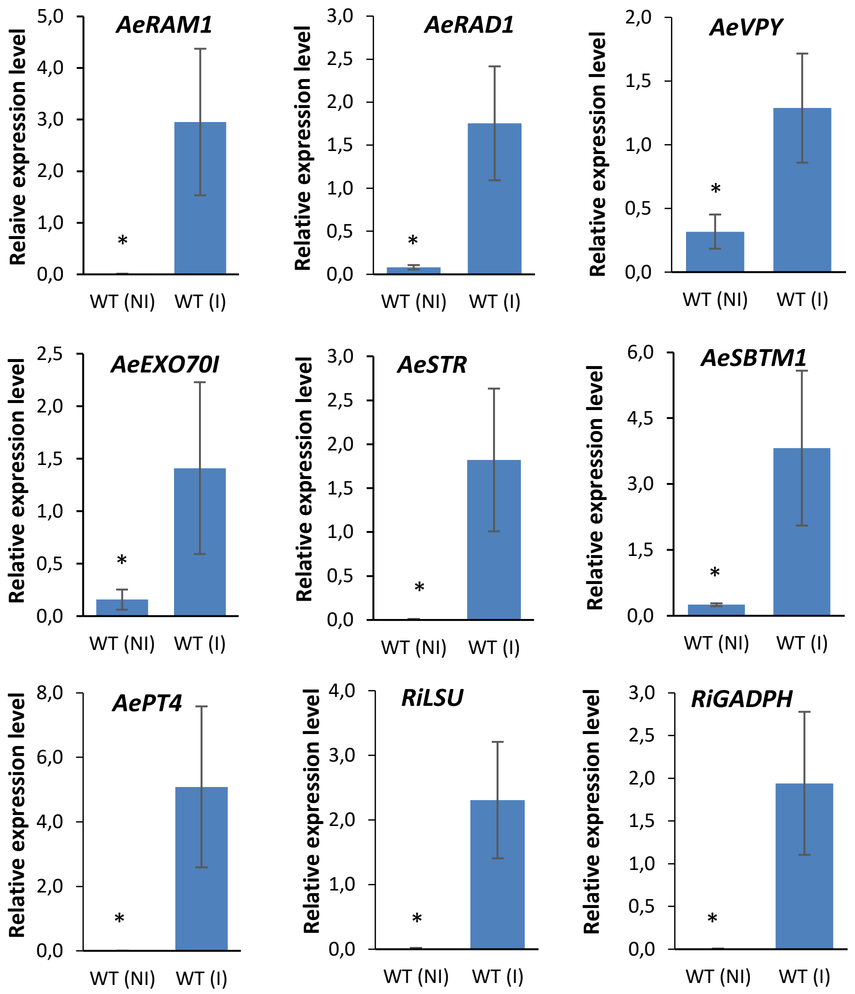

Figure S17. Expression of mycorrhization-related genes in the *A. evenia* wilde type (WT) line. Expression levels of *AeRAM1*, *AeRAD1*, *AeVPY*, *AeEXO70I*, *AeSTR*, *AeSBTM1*, *AePT4*, and of the two fungal genes *RiLSU* and *RiGADPH* was determined by RT-qPCR analysis on wild-type (WT) plant roots cultivated for 8 weeks and inoculated (I) or not (NI) with *R. irregularis*. Expression values were normalized to *AeEF1a* and *Ubiquitin* levels. Means and s.d. were derived from four biological replicates. Asterisks indicate a significant difference when comparing the non-inoculated WT to the inoculated WT according to the Kruskal-Wallis tests (n=4, *P<0.05).
